# Preclinical Development and Assessment of Viral Vectors Expressing a Fusion Antigen of Plasmodium falciparum LSA1 and LSAP2 for Efficacy against Liver-Stage Malaria

**DOI:** 10.1128/IAI.00573-19

**Published:** 2020-01-22

**Authors:** Benedict R. Halbroth, Sarah Sebastian, Ahmed M. Salman, Marta Ulaszewska, Anita Gola, Rhea J. Longley, Chris J. Janse, Shahid M. Khan, Adrian V. S. Hill, Alexandra J. Spencer

**Affiliations:** a The Jenner Institute, University of Oxford, Oxford, United Kingdom; b Leiden University Medical Centre, Leiden, The Netherlands; University of Pennsylvania

**Keywords:** T cells, liver stage, malaria, vaccines

## Abstract

Despite promising progress in malaria vaccine development in recent years, an efficacious subunit vaccine against Plasmodium falciparum remains to be licensed and deployed. Cell-mediated protection from liver-stage malaria relies on a sufficient number of antigen-specific T cells reaching the liver during the time that parasites are present. A single vaccine expressing two antigens could potentially increase both the size and breadth of the antigen-specific response while halving vaccine production costs. In this study, we investigated combining two liver-stage antigens, P. falciparum LSA1 (PfLSA1) and PfLSAP2, and investigated the induction of protective efficacy by coadministration of single-antigen vectors or vaccination with dual-antigen vectors, using simian adenovirus and modified vaccinia virus Ankara vectors. The efficacy of these vaccines was assessed in mouse malaria challenge models using chimeric P. berghei parasites expressing the relevant P. falciparum antigens and challenging mice at the peak of the T cell response. Vaccination with a combination of the single-antigen vectors expressing PfLSA1 or PfLSAP2 was shown to improve protective efficacy compared to vaccination with each single-antigen vector alone. Vaccination with dual-antigen vectors expressing both PfLSA1 and PfLSAP2 resulted in responses to both antigens, particularly in outbred mice, and most importantly, the efficacy was equivalent to that of vaccination with a mixture of single-antigen vectors. Based on these promising data, dual-antigen vectors expressing PfLSA1 and PfLSAP2 will now proceed to manufacturing and clinical assessment under good manufacturing practice (GMP) guidelines.

## Abstract (precis)

Despite promising progress in malaria vaccine development in recent years, an efficacious subunit vaccine against Plasmodium falciparum remains to be licensed and deployed. Cell-mediated protection from liver-stage malaria relies on a sufficient number of antigen-specific T cells reaching the liver during the time that parasites are present. A single vaccine expressing two antigens could potentially increase both the size and breadth of the antigen-specific response while halving vaccine production costs.

## INTRODUCTION

Malaria is a mosquito-borne infectious disease caused by parasitic protozoa belonging to the genus *Plasmodium*. Six *Plasmodium* species and subspecies infect humans and cause one of the most important and life-threatening diseases worldwide: Plasmodium falciparum (the deadliest species), P. vivax, P. ovale *curtisi*, P. ovale *wallikeri*, P. malariae, P. knowlesi. According to the Global Burden of Disease Study, malaria caused approximately 438,000 deaths in 2015 (1), with children under the age of 5 years in sub-Saharan Africa at highest risk. Furthermore, malaria caused more than 200 million clinical episodes in a population of approximately 3.2 billion people living in regions where there is risk of infection.

Apart from the frightful humanitarian impact of the disease, malaria also causes massive economic and social burdens on countries where the disease is endemic. Therefore, there is an urgent need for improved strategies to control malaria, such as novel medication (considering the problem of growing antimalarial-drug resistance), mosquito control, or the development of a highly effective vaccine. To date, the most efficacious vaccine strategies in humans have targeted the preerythrocytic stage of malaria. This has been achieved by induction of high-titer antibodies to block parasite invasion and/or development within the liver with the protein-based vaccine RTS,S (2), induction of malaria-specific T cells with virus-vectored vaccines (3–5), or a combination of antibodies and T cells induced by immunization with irradiated sporozoites (6) or sporozoite administration under drug cover (7).

Antigen (Ag)-specific CD8^+^ T cells have been shown to play a major role in mediating protective immunity against preerythrocytic stages in mice (8–10). We recently demonstrated that protection is dependent on inducing a sufficient number of T cells in the liver to locate and kill the small number of infected hepatocytes in the short window when parasites are present (11). For single-antigen vaccine platforms, it has only been with the development of viral vectors that vaccination-induced T cell responses have been sufficiently high in humans to confer some level of protection from mosquito bite challenge (12). The most advanced virus-vectored vaccine in clinical development is based on a simian adenovirus (Ad) (chimpanzee adenovirus [ChAd]) prime and modified vaccinia virus Ankara (MVA) boost regimen, with both vectors encoding ME-TRAP, a multiple-epitope (ME) string fused to P. falciparum thrombospondin-related adhesion protein (TRAP), a protein of sporozoites (13). Using a prime-boost vaccination regimen, 21% efficacy against infection with P. falciparum sporozoites could be achieved in naive adults (3), with higher (67%) efficacy against natural infection in semi-immune adults (4).

Despite this encouraging progress, even higher levels of protective efficacy have to be achieved to justify mass deployment of such a vaccine. To increase the immunogenicity and efficacy of virus-vectored liver-stage malaria vaccines, different approaches could be employed, for example, including multiple parasite antigens in the viral vector to increase the breadth and number of malaria-specific T cells (14) or incorporating a molecular adjuvant in the viral vector to increase the overall size of the antigen-specific response (15). The latter approach been a rather difficult challenge, but truncated and xenogenized versions of the major histocompatibility complex (MHC) class II invariant chain as a molecular adjuvant have shown promising results in mice (16).

In this study, we explored the possibility of combining multiple antigens in virus-vectored vaccination approaches to increase protective immune responses. To date, only a few candidate liver-stage malaria antigens targeting preerythrocytic stages, other than P. falciparum CSP (PfCSP) or PfTRAP, have been tested extensively (17). As many more parasite proteins have been identified using whole-genome analyses, better candidate antigens could be identified. We recently analyzed viral vectors encoding a number of different sporozoite/liver-stage proteins and compared their immunogenicities and efficacies to those of viral vectors expressing P. falciparum circumsporozoite protein (CSP), the antigen targeted by RTS,S vaccination, and PfTRAP (18). Two of the antigens tested, P. falciparum liver-stage antigen 1 (LSA1) and P. falciparum liver-stage-associated protein 2 (LSAP2), were capable of conferring higher levels of protection than PfCSP or PfTRAP when mice were challenged with chimeric P. berghei parasites expressing the cognate P. falciparum antigen. In this study, therefore, we assessed the immunogenicity and efficacy of combining LSA1 and LSAP2 in virus-vectored vaccination approaches. To maximize the potential efficacy and immunogenicity of the vaccines, we also included the molecular adjuvant shark Ii chain transmembrane (TM) domain in the viral vector. Single- and dual-antigen-expressing viral vectors were generated and assessed in terms of their immunogenicities and protective efficacies in inbred and outbred mouse strains with the overall aim of selecting the most promising vaccine candidate to manufacture to clinical grade (under good manufacturing practice [GMP] guidelines) for use in human clinical trials.

## RESULTS

### Combinations of LSA1 and LSAP2 with PfTRAP.

To determine whether combinations of two novel liver-stage antigens (PfLSA1 and PfLSAP2) (18) with PfTRAP, the most advanced liver-stage T cell antigen, could improve protection, mice were vaccinated with a heterologous ChAd type 63 (ChAd63)-MVA prime-boost vaccination regimen and challenged with double-chimeric P. berghei parasites expressing either PfTRAP and PfLSA1 or PfTRAP and PfLSAP2 10 days after MVA boost, corresponding to the peak of CD8^+^ T cell responses. In each experiment, mice were vaccinated either with a single viral vector targeting one antigen or with two viral vectors targeting two different antigens. When mice were vaccinated with two viral vectors, the vectors were either administered into separate legs or mixed together prior to intramuscular injection, and the antigen dose was kept constant, and therefore, the mice received twice the total amount of virus but the same amount of antigen. Blood samples were taken 7 days after MVA boost to determine immunogenicity against each antigen prior to sporozoite challenge 10 days after MVA boost. Since gamma interferon (IFN-γ) is a critical cytokine required for parasite clearance (19), antigen-specific cells were identified by intracellular staining for IFN-γ following peptide stimulation. As vaccination with viral vectors targeting two antigens could potentially result in antigenic competition and a decrease in immunogenicity against either antigen (20, 21), we characterized immunogenicity, as well as efficacy, in these studies.

**FIG 1:**
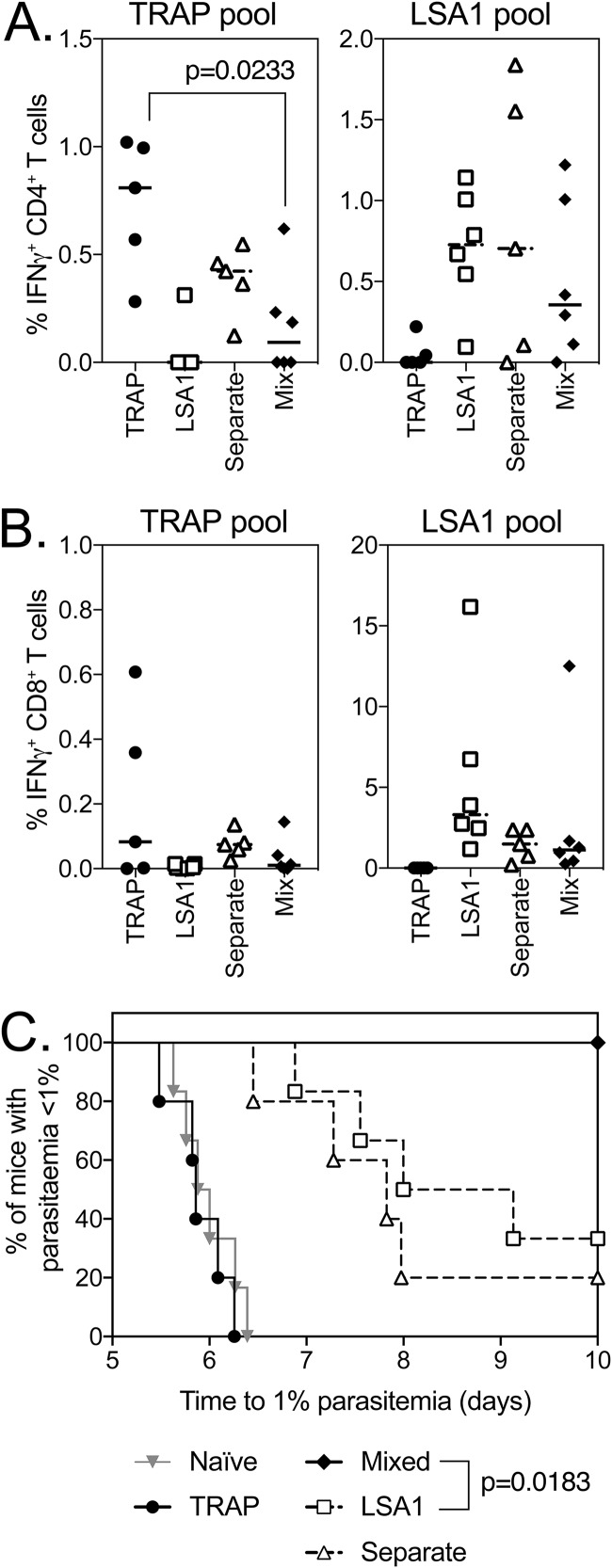
Immunogenicity and efficacy of coadministration of PfTRAP and PfLSA1. (A and B) BALB/c mice (6 per group) were vaccinated with 10^8^ IU ChAd63, followed 7 weeks later with a 10^6^-IU MVA boost of each vaccine expressing PfTRAP (circles), PfLSA1 (squares), both vaccines administered in separate legs (open triangles), or both vaccines mixed (diamonds). One week post-MVA boost (week 7), a blood sample was taken, and PBMCs were analyzed by ICS after stimulation with a PfTRAP or PfLSA1 peptide pool. Ten days after MVA boost, the mice were challenged with 1,000 chimeric P. berghei sporozoites expressing P. falciparum PfTRAP and PfLSA1 and monitored for development of blood-stage malaria. The graphs represent the frequency of blood CD4^+^ IFN-γ^+^ (A) or CD8^+^ IFN-γ^+^ (B) T cells. (C) Time to reach 1% parasitemia plotted on a Kaplan-Meier survival curve. The data points indicate individual mice, and the horizontal lines show the median response per group.

Following coadministration of ChAd63 and MVA vaccines expressing PfTRAP and PfLSA1, IFN-γ-producing CD4^+^ (Fig. 1A) and CD8^+^ (Fig. 1B) T cells were observed in the blood, with a statistically significant decrease observed only in PfTRAP-specific CD4^+^ T cells in the coadministration group, although slight but nonsignificant decreases in CD8^+^ T cell responses to PfTRAP and PfLSA1 were also observed (Fig. 1B). However, slight decreases in immunogenicity did not compromise efficacy, as coadministering vaccines resulted in 100% sterile protection from challenge with P. berghei parasites expressing both PfTRAP and PfLSA1, with a statistically significant enhancement in survival compared to mice vaccinated only with PfLSA1 (30% sterile efficacy) or PfTRAP-expressing vaccines (0% sterile efficacy and no delay in prepatency compared to naive controls) (Fig. 1C). Interestingly, this was observed only when vaccines were mixed and coadministered, as no increase in efficacy was observed when PfTRAP and PfLSA1 were injected into separate legs (Fig. 1C). In a separate experiment, antigen-specific responses in the liver, inguinal draining lymph nodes, and spleen were compared to determine whether there was an underlying immunological difference driving this increase in efficacy when vaccines were coadministered as injections into separate legs or mixed together, but no differences could be observed between groups (see Fig. S1 in the supplemental material). The increase in vaccine efficacy through coadministration was reliant on a base level of efficacy of the single-antigen vaccines, as when PfTRAP was mixed with PfFalstatin, another P. falciparum Ag shown to induce a small delay in time to parasitemia, no difference in survival relative to the naive mice was observed with either single or coadministered vaccines (see Fig. S2 in the supplemental material).

**FIG 2:**
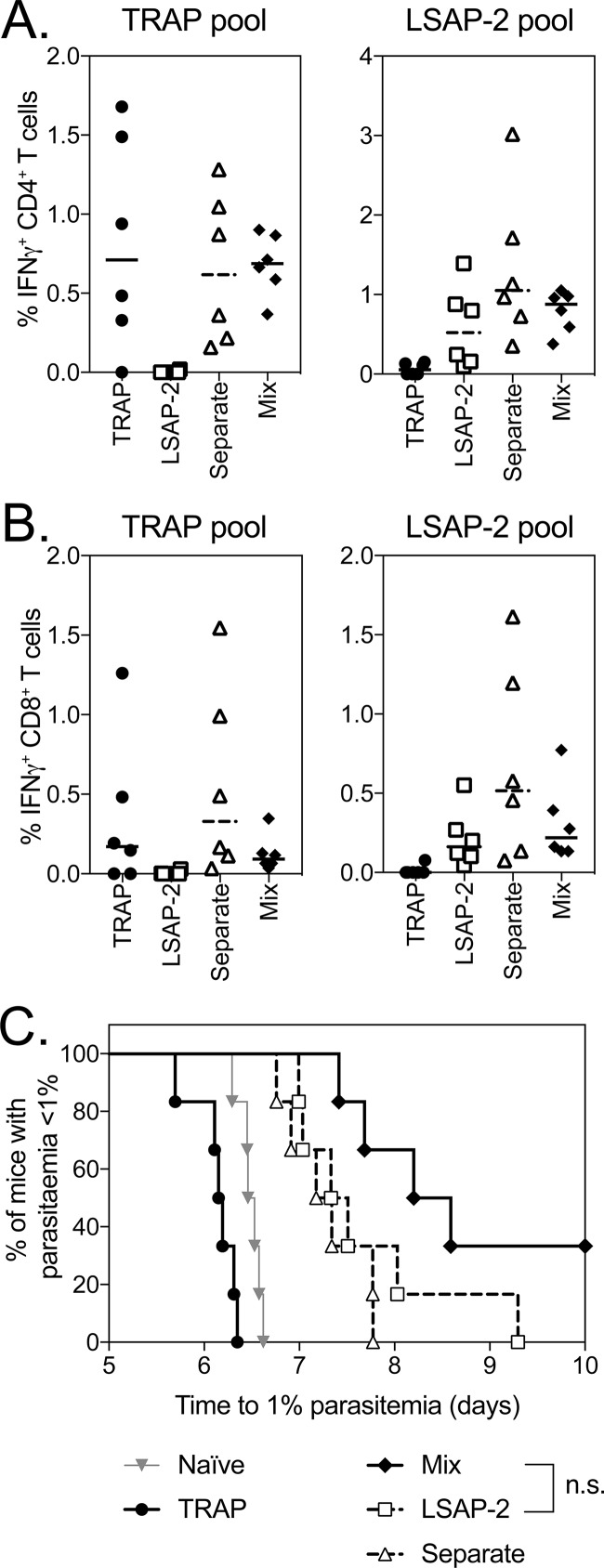
Immunogenicity and efficacy of coadministration of PfTRAP and PfLSAP2. (A and B) BALB/c mice (6 per group) were vaccinated with 10^8^ IU ChAd63 followed 7 weeks later with a 10^6^-IU MVA boost of each vaccine expressing PfTRAP, PfLSAP2, both vaccines administered in separate legs, or both vaccines mixed. One week post-MVA boost (week 7), a blood sample was taken, and PBMCs were analyzed by ICS after stimulation with a PfTRAP or PfLSAP2 peptide pool. Ten days after MVA boost, the mice were challenged with 1,000 chimeric P. berghei sporozoites expressing PfTRAP and PfLSAP2 and monitored for development of blood-stage malaria. The graphs represent the frequency of blood CD4^+^ IFN-γ^+^ (A) or CD8^+^ IFN-γ^+^ (B) T cells. (C) Time to reach 1% parasitemia plotted on a Kaplan-Meier survival curve. The data points indicate individual mice, and the horizontal lines show the median response per group.

When the coadministration of ChAd63 and MVA vaccines expressing PfTRAP and PfLSAP2 was assessed, equivalent immune responses to PfTRAP and PfLSAP2 of both IFN-γ-producing CD4^+^ (Fig. 2A) and CD8^+^ (Fig. 2B) T cells were observed in the blood of mice 7 days after MVA boost. Consistent with previous studies, vaccination with PfLSAP2 increased from challenge with P. berghei expressing PfTRAP and PfLSAP compared to PfTRAP-only-vaccinated animals (Fig. 2C), with coadministration of PfTRAP and PfLSAP2 leading to the highest efficacy level, as shown by 40% sterile efficacy and a delay in prepatency when the vaccines were mixed together, although this increase in survival was not statistically significant compared to mice vaccinated with only PfLSAP2. Again, when the vaccines were administered into separate legs, no effect on efficacy was observed compared to mice vaccinated with only a single antigen.

### Combined administration of PfLSA1 and PfLSAP2.

**FIG 3:**
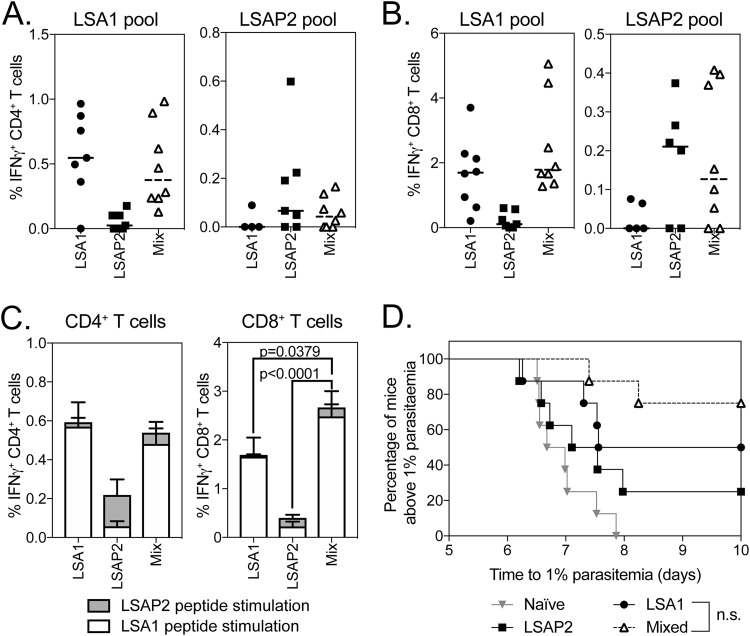
Immunogenicity and efficacy of coadministration of PfLSA1 and PfLSAP2. (A to C) BALB/c mice (8 per group) were vaccinated with 10^8^ IU ChAd63 expressing PfLSA1, PfLSAP2, or both vaccines mixed (Mix), resulting in a total virus dose of 2 × 10^8^ IU. A blood sample was taken on day 14, and PBMCs were analyzed by ICS after stimulation with a PfLSA1 or PfLSAP2 peptide pool. At day 17 postvaccination, the mice were challenged with 1,000 chimeric P. berghei sporozoites expressing PfLSA1 and PfLSAP2 and monitored for development of blood-stage malaria. The graphs represent the frequency of blood CD4^+^ IFN-γ^+^ (A) or CD8^+^ IFN-γ^+^ (B) T cells or summed CD4^+^ IFN-γ^+^ or CD8^+^ IFN-γ^+^ T cell responses (C). The bars represent median response; error bars indicate standard error of the mean. (D) Time to reach 1% parasitemia plotted on a Kaplan-Meier survival curve. The data points indicate individual mice, and the horizontal lines show the median response per group.

Having found an additive effect on efficacy when combining PfTRAP with either PfLSA1 or PfLSAP2, we investigated whether the combination of PfLSA1 with PfLSAP2 could increase efficacy against P. berghei parasites expressing both PfLSA1 and PfLSAP2 compared to vaccines targeting the single antigens. In an initial prime-boost experiment, we were unable to detect an effect of combining the antigens, as immunization with a single antigen vector expressing PfLSA1 or PfLSAP2 conferred high protective efficacy (>80%) (see Fig. S3A in the supplemental material). In order to be able to measure differences in efficacy, we chose to test efficacy by challenging mice after a single ChAd63 vaccination. BALB/c mice were vaccinated with 10^8^ infectious units (IU) of ChAd63 expressing either PfLSA1 or PfLSAP2, and another group (mixed) was coadministered a full dose of both vaccines mixed prior to administration. Two weeks postimmunization, at the peak of the immune response (22), and prior to challenge with double-chimeric P. berghei parasites [Pb(PfLSA1 + PfLSAP2), a blood sample was analyzed for immunogenicity]. Encouragingly, while all the aive control mice developed blood-stage infections, 25% (2/8), 50% (4/8), and 75% (6/8) of the mice vaccinated with PfLSA1, PfLSAP2, or both vaccines (mixed) were protected (Fig. 3D). A statistically significant difference was observed in the length of the prepatent period, with mice vaccinated with PfLSA1 (*P* < 0.05) and mixed-vaccination mice (*P* < 0.001) having longer prepatent periods than the naive control group (Fig. 3D). There was no significant difference in prepatent periods between the PfLSA1 and mixed groups of mice. Importantly, there was no statistically significant difference in IFN-γ-producing CD4^+^ (Fig. 3A) or CD8^+^ (Fig. 3B) T cells between single- and mixed-vaccination groups. Interestingly, the total immune response detected in mixed-vaccination mice was dominated by a response to the PfLSA1 peptide pool (Fig. 3C). While equivalent total levels of antigen-specific cells were observed for CD4^+^ T cells (Fig. 3C, left), mixing PfLSA1 and PfLSAP2 led to a significant increase in the overall frequency of antigen-specific CD8^+^ T cell responses compared to each antigen alone (Fig. 3C, right). Even with highly immunogenic antigens, protection following Ad-MVA prime boost vaccination wanes with time, corresponding to a decrease in the number of antigen-specific T cells (23). To determine whether combining the two antigens would confer longer-lasting efficacy, CD1 mice were vaccinated with ChAd63-MVA vectors expressing PfLSA1, PfLSAP2, or both vaccines (mixed) and were challenged with double-chimeric P. berghei parasites [Pb(PfLSA1 + PfLSAP2)] 5 months after the MVA boost. Despite a small significant increase in survival compared to naive controls when the mice were vaccinated with PfLSAP2 or mixed vaccines, there was no significant effect of mixed vaccination compared to single-antigen controls (see Fig. S3B).

### Comparison of immunogenicities of single- and dual-antigen-containing vectors.

Based on the observed improvement in protective immunity by mixing vectors containing either PfLSA1 or PfLSAP2, we set about generating clinically relevant viral vectors expressing both antigens (dual-fusion antigen vectors). For this purpose, we used ChAdOx1, a relatively new simian adenoviral vector with low-seroprevalence in humans (24) that has been used in a number of recent clinical trials (12). Importantly, equivalent levels of IFN-γ-producing CD4^+^ and CD8^+^ T cells were induced by vaccination with single-antigen ChAdOx1 vectors that expressed either PfLSA1 or PfLSAP2 and by ChAd63 vectors (see Fig. S4 in the supplemental material).

To further maximize T cell responses, we chose to incorporate in the ChAdOx1 vectors the recently described shark and trout Ii chain TM domains, which we have shown to significantly enhance antigen-specific CD8^+^ T cell responses after immunization with ME-TRAP-expressing viral vectors (16). We therefore generated the following ChAdOx1 vectors: single-antigen vectors expressing either PfLSA1 or PfLSAP2 and a dual-fusion antigen ChAdOx1 vector expressing a fusion (PfLSA1-PfLSAP2) (see Fig. S5 in the supplemental material). They were fused to the trout Ii chain, the TM domain of the trout Ii chain, the shark Ii chain, or the TM domain of the shark Ii chain. These vectors were assessed in three separate experiments for their immunogenicities. Although no significant increase in CD4^+^ or CD8^+^ T cells was observed for any single molecular adjuvant, single-antigen vectors containing the TM domain of the shark Ii chain showed a trend toward an increase in antigen-specific CD8^+^ T cells. Given the small size of the shark Ii chain TM and its low sequence homology to the human Ii chain TM, we chose to use this domain in the single- and dual-fusion antigen vectors.

**FIG 4:**
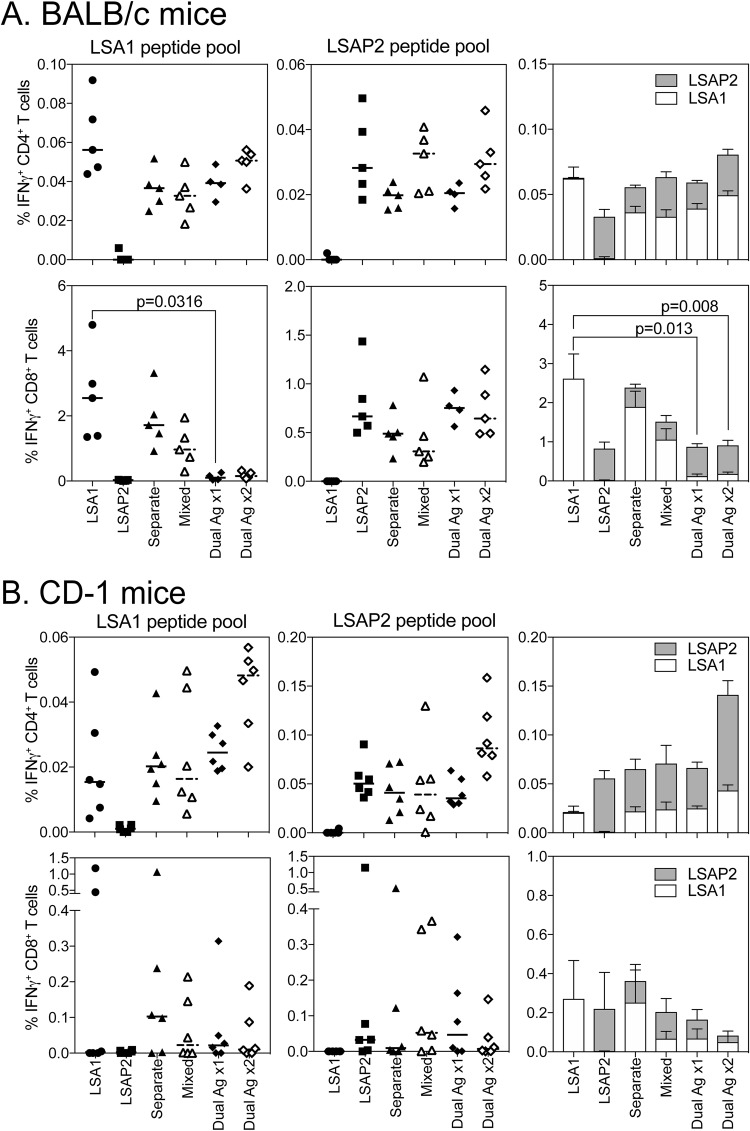
Immunogenicity of dual-antigen-expressing ChAdOx1 in inbred and outbred mice. (A) BALB/c mice (5 per group) were immunized with 10^8^ IU of each ChAdOx1 vector expressing either PfLSA1, PfLSAP2, both vectors administered into separate legs (Separate), both vectors premixed and injected into both legs (Mixed), or dual-antigen-expressing vector (Dual Ag ×1) or 2 × 10^8^ IU of the dual-antigen-expressing vector (Dual Ag ×2). T cell responses to PfLSA1 or PfLSAP2 peptide pools were analyzed by ICS. The percentages of blood CD4^+^ and CD8^+^ T cells positive for IFN-γ are shown. The single points represent individual mice; the horizontal lines denote the median response per group. To determine if there was a difference between the total antigen-specific response, CD4^+^ or CD8^+^ IFN-γ^+^ responses to each antigen were summed, and the data were analyzed with a two-way repeated-measures ANOVA with *post hoc* positive effect to determine the effect of vaccine for each T cell subset. The *P* values denote the levels of significance observed. The bars represent median response; error bars indicate standard error of the mean. (B) CD1 mice (6 per group) were immunized with 10^8^ IU ChAdOx1 vectors as for panel A, with spleens harvested 2 weeks later. T cell responses to PfLSA1 and PfLSAP2 peptide pools were analyzed by ICS. The percentages of CD4^+^ and CD8^+^ T cells positive for IFN-γ are shown. To determine if there was a difference between the total antigen-specific response, CD4^+^ or CD8^+^ IFN-γ^+^ responses to each antigen were summed, and the data were analyzed with a two-way repeated-measures ANOVA, but no significant effect was observed for either T cell subset or vaccine.

To compare the immunogenicities of single- and dual-fusion antigen vectors, BALB/c mice were immunized with 10^8^ IU of ChAdOx1 expressing sharkTM/Ii-LSA1 or sharkTM/Ii-LSAP2, with equal amounts of either single-antigen vaccine administered into separate legs or mixed together and split between two legs. The dual-fusion antigen ChAdOx1 vector sharkTM/Ii-PfLSA1-PfLSAP2 was administered at a dose of 1 × 10^8^ IU or 2 × 10^8^ IU to account for the increase in the total amount of virus when two single-antigen vectors were administered. The PfLSA1- and PfLSAP2-specific CD4^+^ T cell responses (Fig. 4A, top) and PfLSAP2-specific CD8^+^ T cell responses (Fig. 4A, bottom) of all the groups were similarly strong. Vaccination with the dual-fusion antigen vector sharkTM/Ii-LSA1-LSAP2 only marginally increased T cell responses. Interestingly, PfLSA1-specific CD8^+^ T cell responses were significantly reduced in mice vaccinated with the dual-fusion antigen vector compared to single-antigen controls (Fig. 4A, bottom). Doubling the dose of the dual-fusion antigen vector sharkTM/Ii-LSA1-LSAP2 did not compensate for the reduction in PfLSA1-specific CD8^+^ T cell immunogenicity. To determine whether this was an effect of MHC restriction, immunogenicity in outbred CD-1 was assessed. PfLSA1 and PfLSAP2 CD4^+^ T cell responses were low, with a trend toward the highest immunogenicity in mice vaccinated with 2 × 10^8^ IU of the dual-fusion antigen vector sharkTM/Ii-PfLSA1-PfLSAP2 (Fig. 4B). PfLSA1- and PfLSAP2-specific CD8^+^ T cell responses in outbred mice were lower and more variable than those of inbred BALB/c mice, and overall, there was no significant decrease in IFN-γ-producing CD8^+^ PfLSA1- or PfLSAP2-specific T cells or the total size of the CD8^+^ T cell antigen-specific response when the dual-fusion antigen vector sharkTM/Ii-PfLSA1-PfLSAP2 was used compared to the single-antigen vaccines (Fig. 4B, bottom).

### Immunogenicity and efficacy of the prime-boost vaccination approach of dual-antigen-expressing vectors.

Having established that ChAdOx1 vectors were immunogenic, we then tested protective efficacy induced by vaccination using the highly immunogenic ChAdOx1 prime and MVA boost regimen, which would be deployed in a clinical trial. Consistent with previous data (16), incorporation of the shark Ii chain TM domain in the vectors did not enhance either the CD4^+^ or CD8^+^ T cell response following a single immunization with the “adjuvanted” MVA vectors (see Fig. S6 in the supplemental material). In addition, when mice were boosted with adjuvanted or nonadjuvanted MVAs, only unadjuvanted MVAs were capable of boosting the PfLSA1-specific responses in both inbred (see Fig. S6A) and outbred (see Fig. S6B) mice. Therefore, for efficacy assessment, all ChAdOx1 vectors expressed shark TM Ii fusion antigens, but mice were boosted only with the relevant unadjuvanted PfLSA1-, PfLSAP2-, or PfLSA1-PfLSAP2 fusion-expressing MVA vector.

**FIG 5:**
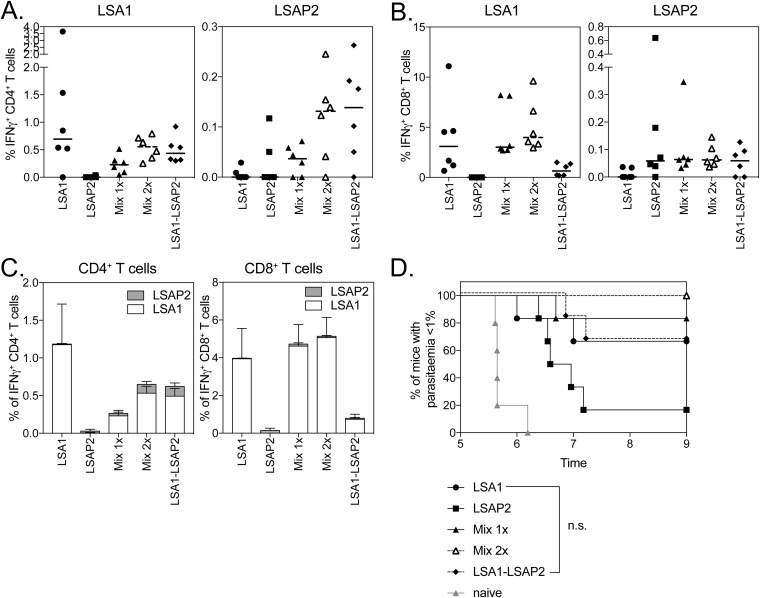
Immunogenicity and efficacy of dual-antigen-expressing vectors in BALB/c mice following prime-boost vaccination. (A to C) BALB/c mice (6 per group) were immunized with 10^8^ IU of ChAdOx1 (the Mix 2× group received 2 × 10^8^ IU total virus) and boosted 6 weeks later with 10^7^ PFU MVA (the Mix 2× group received 2 × 10^7^ PFU total virus), with antigen inserts as indicated on the *x* axes. In ChAdOx1 vectors, the antigen was fused to the adjuvant sharkTM/Ii; in MVA vectors, the antigen was fused to tPA. A blood sample was taken in week 7 and analyzed by ICS after stimulation with a PfLSA1 (A) or PfLSAP2 (B) peptide pool. The graphs represent the frequency of PfLSA1-specific (A) or PfLSAP2 (B) CD4^+^ IFN-γ^+^ or CD8^+^ IFN-γ^+^ T cells or the summed CD4^+^ I FN-γ^+^ or CD8^+^ IFN-γ^+^ T cell responses (C). Mice were challenged with 1,000 chimeric P. berghei sporozoites expressing PfLSA1 and PfLSAP2 and monitored for development of blood-stage malaria. The bars represent median response; error bars indicate standard error of the mean. (D) Time to reach 1% parasitemia plotted on a Kaplan-Meier survival curve. All the groups showed significant increases in survival compared to naive controls above the Bonferroni-corrected threshold (*P* < 0.003), but no significance (n.s.) between vaccinated groups was observed.

In an initial experiment, BALB/c mice were vaccinated with ChAdOx1 and MVA vectors, and 1 week post-MVA boost, the frequencies of CD4^+^ T cells against PfLSA1 were similar between all study groups that received a vector expressing PfLSA1 but highest in mice that were vaccinated with vectors expressing PfLSA1 only (Fig. 5A). As seen in previous experiments (Fig. 4A), the PfLSA1-specific CD8^+^ T cell response was lower in mice immunized with the dual-antigen vectors expressing the PfLSA1-PfLSAP2 fusion compared to mice vaccinated with single-antigen vectors expressing PfLSA1 (Fig. 5B). The immune response in BALB/c mice was also dominated by a response to PfLSA1, with PfLSAP2-specific T cell responses generally lower (Fig. 5C) but with no significant differences between groups. When challenged with 1,000 Pb(PfLSA1 + PfLSAP2) sporozoites, the prepatent period was significantly delayed in all vaccinated mice compared to naive mice (Fig. 5D), with high levels of efficacy in all the groups vaccinated with a vaccine containing PfLSA1. While vaccination with a dual-fusion antigen vector expressing PfLSA1-PfLSAP2 resulted in a slight drop in T cell responses, there was no significant difference between the times for the mice to reach 1% parasitemia and those for PfLSA1-vaccinated mice (Fig. 5D).

**FIG 6:**
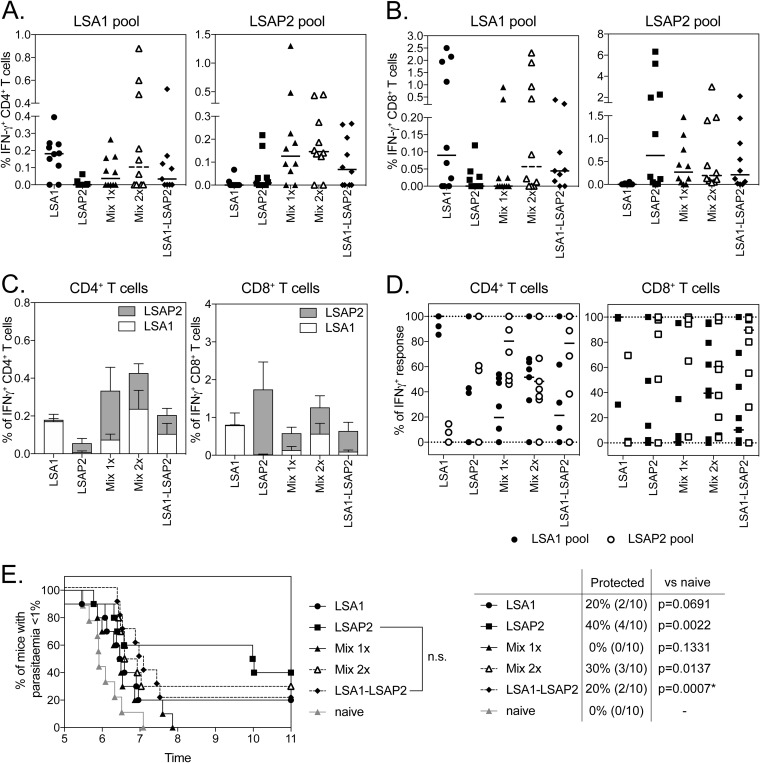
Immunogenicities and efficacies of dual-antigen-expressing vectors in outbred mice. (A to D) CD-1 mice (10 per group) were immunized with 10^8^ IU of ChAdOx1 (The Mix 2× group received 2 × 10^8^ IU total virus) and boosted 6 weeks later with 10^7^ PFU MVA (the Mix 2× group received 2 × 10^7^ PFU total virus), with antigen inserts as indicated on the *x* axes. In ChAdOx1 vectors, the antigen was fused to the adjuvant sharkTM/Ii; in MVA vectors, the antigen was fused to tPA (sharkTM/Ii → tPA). A blood sample was taken in week 7 and analyzed by ICS after stimulation with a PfLSA1 (A) or PfLSAP2 (B) peptide pool. The graphs represent the frequency of CD4^+^ IFN-γ^+^ (A) or CD8^+^ IFN-γ^+^ (B) PfLSA1 or PfLSAP2 T cells, summed CD4^+^ IFN-γ^+^ or CD8^+^ IFN-γ^+^ T cell response (C), or the percentage of CD4^+^ IFN-γ^+^ or CD8^+^ IFN-γ^+^ T cells specific for either PfLSA1 or PfLSAP2 (D). The mice were challenged with 1,000 chimeric P. berghei sporozoites expressing PfLSA1 and PfLSAP2 and monitored for development of blood-stage malaria. (E) Time to 1% parasitemia plotted on a Kaplan-Meier survival curve. Only PfLSAP2 and PfLSA1-LSAP2 showed a significant increase in survival compared to naive controls above the Bonferroni-corrected threshold (*P* < 0.003), but no significance between vaccinated groups was observed. The error bars indicate standard deviations.

**FIG 7:**
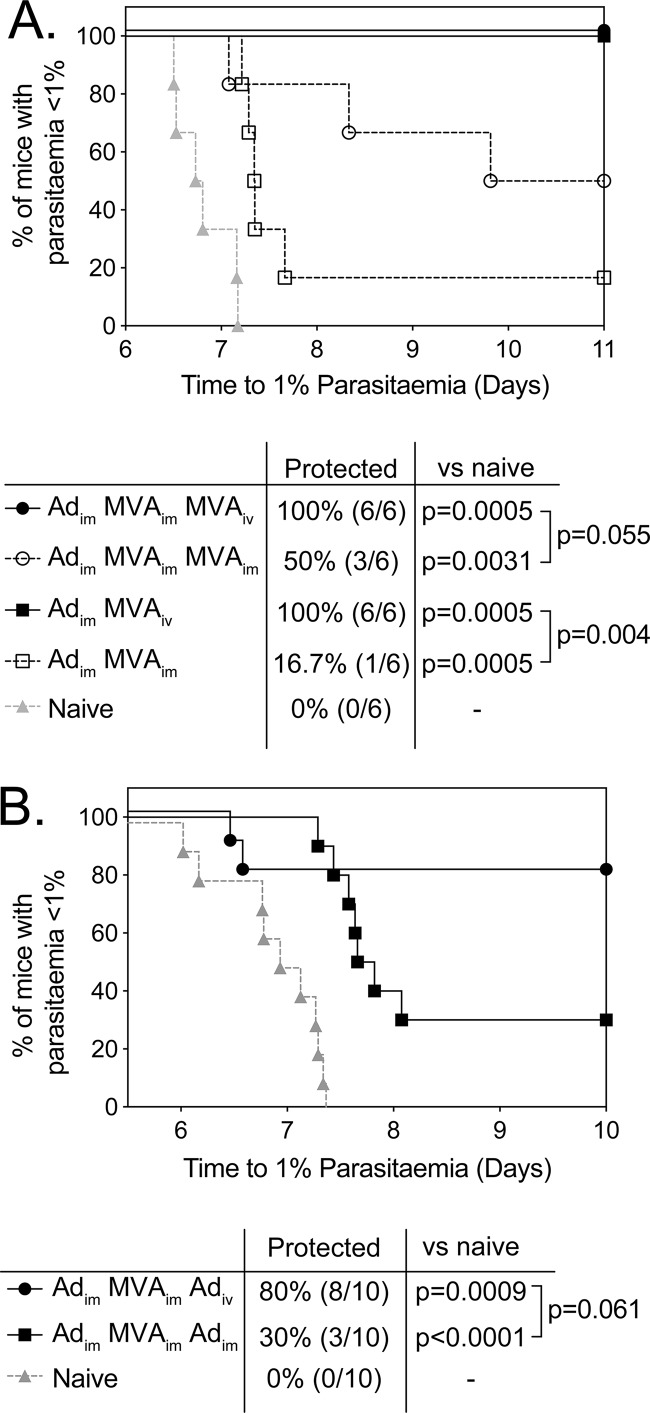
Prime-target immunization improves the efficacy of the dual LSA1-LSAP2-expressing vectors. (A) BALB/c mice (6 per group) were immunized with 10^8^ IU of ChAdOx1.LSA1-LSAP2 (i.m.), boosted 2 weeks later with 10^6^ PFU MVA.LSA1-LSAP2 (i.m.), and targeted a further 2 weeks later with 10^7^ PFU MVA.LSA1-LSAP2 (i.v. or i.m.). Alternatively, mice were primed with 10^8^ IU of ChAdOx1.LSA1-LSAP2 (i.m.) and targeted 2 weeks later with 10^7^ PFU MVA.LSA1-LSAP2 (i.v. or i.m.). The mice were challenged with 1,000 chimeric P. berghei sporozoites expressing PfLSA1 and PfLSAP2 3 weeks after the targeting immunization and monitored for development of blood-stage malaria. The time to reach 1% parasitemia is plotted on a Kaplan-Meier survival curve. All the groups showed significant increases in survival compared to naive controls above the Bonferroni-corrected threshold (*P* < 0.008), with a significant difference observed between Ad_im_-MVA_im_ and Ad_im_-MVA_iv_. (B) CD-1 mice (10 per group) were immunized with 10^8^ IU of ChAdOx1.LSA1-LSAP2 (i.m.), boosted 4 weeks later with 10^6^ PFU MVA.LSA1-LSAP2 (i.m.), and targeted a further 2 weeks later with 10^9^ IU ChAdOx1.LSA1-LSAP2 (i.m.). The mice were challenged with 1,000 chimeric P. berghei sporozoites expressing PfLSA1 and PfLSAP2 3 weeks after the final immunization and monitored for development of blood-stage malaria. The time to reach 1% parasitemia is plotted on a Kaplan-Meier survival curve. Both groups showed significant increases in survival compared to naive controls above the Bonferroni-corrected threshold (*P* < 0.017), but a statistically significant difference between targeting of mice with Ad administered i.v. or i.m. was not observed.

Immunogenicities and efficacies of PfLSA1-, PfLSAP2-, and PfLSA1-LSAP2-expressing vectors were investigated in a 6-week prime-boost ChAdOx1-MVA vaccination regimen in CD1 mice (Fig. 6). No significant difference in CD4^+^ (Fig. 6A) and CD8^+^ (Fig. 6B) T cell responses to either PfLSA1 or PfLSAP2 were observed between any of the vaccination groups. Importantly vaccination with a mixture of the two antigens or dual-fusion antigen-expressing vector did not lead to domination of the immune response to a single antigen, as the frequencies (Fig. 6C) and proportions (Fig. 6D) of PfLSA1 or PfLSAP2 CD4^+^ or CD8^+^ T cell responses were equivalent across groups. In contrast to previous experiments, vaccinating mice with the single-antigen virus expressing PfLSAP2 conferred the highest level of sterile efficacy (4/10; *P* < 0.01), although the time to patency was not significantly different from that of naive controls (Fig. 6E). However, mice vaccinated with the dual-fusion antigen vectors were the only group of animals to show a significant increase in the time to patency compared to naive controls (Fig. 6E). Most importantly, when we assessed the efficacy of dual-fusion antigen-expressing ChAdOx1 and MVA vectors in a prime-target regimen aimed at targeting CD8^+^ T cells to the liver (25) by intravenous administration of the viral vector, higher levels of sterile efficacy were observed following intravenous MVA (Fig. 7A) or ChAdOx1 (Fig. 7B) administration and challenging mice 3 weeks after the targeting vaccination.

In summary, expression of the fusion antigen PfLSA1-PfLSAP2 in viral vectors is capable of inducing broad T cell responses in outbred mice, with efficacy equivalent to that of vaccination with single antigens.

## DISCUSSION

Preerythrocytic subunit malaria vaccines in clinical development are based on a small handful of historical candidate antigens, mainly PfCSP and PfTRAP, but success in clinical trials has been limited. As evidence suggests that antigens other than PfCSP and PfTRAP contribute to protective immunity (26–29), it is likely that the inclusion of multiple antigens in a vaccine is necessary to reach sufficient levels of protection. Encouragingly, with science progressing into the omics era, many more potential liver-stage vaccine antigen candidates have been identified from the parasite’s genome.

Preclinical assessment of these antigens has been difficult due to the fact that P. falciparum (which causes the highest malaria burden in humans) does not naturally infect standard laboratory animals. However, this can be partially overcome by challenging mice with a murine parasite strain (P. berghei) that expresses the relevant P. falciparum vaccine antigen as a transgene (18). This is especially useful when there is no known orthologue of the P. falciparum vaccine antigen present in the murine *Plasmodium* species.

Previous work from our group using this chimeric P. berghei challenge model has shown that viral vectors encoding PfLSA1 or PfLSAP2 induce high levels of efficacy in mice (18). Both PfLSA1 and PfLSAP2 are expressed in developing merozoites either inside the parasitophorous vacuole or as part of the parasitophorous vacuole membrane (PVM) (30). PfLSA1 is also known to be highly conserved between P. falciparum strains (31), essential for late liver-stage development (32), and the target of cellular immunity after natural infection (33–36) or vaccination with irradiated sporozoites (37). Therefore, we wished to test the combination of these antigens together with PfTRAP, the leading antigen in viral vectors that has shown some level of efficacy in humans (3, 4), or with each other.

Initial experiments tested the immunogenicity and efficacy of coadministration of ChAd63 and MVA vectors. Despite theoretical concerns that administration of more than one antigen would result in antigenic competition (20, 21), no detrimental effect was observed when any of these liver-stage vaccine candidate antigens were coadministered. Administration of PfLSA1 and PfLSAP2 with PfTRAP improved the efficacy of prime-boost vaccination compared to single administration of ChAd63 vectors, with a combination of PfLSA1 and PfLSAP2 conferring significantly higher levels of efficacy than a single antigen alone. Interestingly, we observed an increase in efficacy only when the vaccines were mixed and injected together, not when vaccines were administered into separate sites, which is consistent with previous data from when we coadministered PfTRAP with another preerythrocytic antigen, PfUIS3 (38). This observed increase in efficacy with mixed but not separate administration was surprising, as the percentages of effector cells in the blood were equal between groups. As CD8^+^ T cell-mediated protection relies on reaching a threshold of antigen-specific cells in the liver (11), one explanation may be that mixing vaccines and injecting them into two sites may have increased the total number of cells in the liver, which is not reflected by responses in the blood, which measure only the frequency of antigen-specific cells. This is supported by evidence that intravenous administration of MVA (thus increasing the number of sites of T cell activation across the entire mouse) leads to higher CD8^+^ T cell responses in the spleen and liver (25; A. J. Spencer and A. V. S. Hill, unpublished data), in addition to reports of improved immunogenicity of cancer vaccines by increasing the number of injection sites (39). In the absence of human efficacy data with either PfLSA1 or PfLSAP2 antigen from viral vectors and with preclinical data suggesting the two in combination would provide the greatest efficacy, we set about generating vectors that expressed both antigens as a fusion protein.

Although simian adenoviruses have been shown to express relatively large antigenic inserts (40), many combination antigen approaches to date have been hampered by the lack of good promoters or insertions sites. Our initial attempts to encode PfTRAP and PfCSP separately in different loci of an adenoviral vector, or using a bidirectional cytomegalovirus (CMV)-derived promoter, failed to induce strong immunogenicity against both antigens (data not shown). Therefore, a PfLSA1-PfLSAP2 fusion construct, which comprised PfLSAP2 fused to the C terminus of PfLSA1 with a peptide linker separating the two antigens (LSA1-LSAP2), was produced. The encoded antigen—PfLSA1 (461 amino acids [aa]), PfLSAP2 (302 aa), or PfLSA1-LSAP2 (770 aa, including the peptide linker)—was fused to the tPA leader sequence (which is known to improve expression and immunogenicity) (41–43). In addition, we wished to maximize potential immunogenicity by inclusion of a molecular adjuvant, the MHC class II chain, which we have shown to increase CD8^+^ T cells in nonhuman primates (15). Recent optimization work identified shark and trout Ii chain TM domains as optimal for enhancing the T cell response, with the added benefits of small size and minimal homology to the human Ii chain to alleviate some safety concerns (16).

All novel adjuvanted and nonadjuvanted vectors were then analyzed in prime-only and ChAdOx1-MVA prime-boost regimens for immunogenicity, and despite the reduced ability to induce PfLSA1-specific CD8^+^ T cell responses observed when BALB/c mice were vaccinated with a PfLSA1-LSAP2 fusion construct, similar levels of efficacy against parasite challenge were achieved (Fig. 5). Ii chain TM is primarily believed to increase T cell responses by stabilizing and improving protein expression by multimerization of the antigen (16). The ability of the shark Ii TM domain to enhance responses is therefore highly antigen dependent, which would explain the minimal impact of the shark Ii TM on LSA1 and LSAP2 responses (see Fig. S5). Subsequent testing in CD1 mice, an outbred mouse population with a diverse MHC repertoire, resulted in greater variability in vaccine immunogenicity and efficacy but largely confirmed results obtained in BALB/c mice. Although the reduced immunogenicity in outbred mice corresponded with reduced levels of protective efficacy in all groups (Fig. 6), the combination of adjuvanted ChAdOx1 with nonadjuvanted MVA seemed to be the most promising. Most importantly, while inclusion of an additional antigen did slightly reduce the response to each single antigen (Fig. 5 and 6), overall, PfLSA1-PfLSAP2-encoding vectors showed levels of efficacy similar to those of single PfLSA1 or PfLSAP2 vectors (Fig. 5 and 6); therefore, it is possible to include two antigens without compromising efficacy. In addition, when PfLSA1-PfLSAP2 vectors were administered in a prime-target regimen, where the final vector is administered by intravenous injection, higher overall levels of efficacy were observed (Fig. 7). Therefore, should PfLSA1-PfLSAP2 administered by standard vaccination prove efficacious in a controlled human infection study, altering the route of vaccination would be a plausible strategy to enhance vaccine efficacy.

By using the chimeric parasite challenge model, we have been able to ensure that T cell-mediated efficacy is not compromised following vaccination with dual-antigen-expressing vectors in mice. However, there are major limits to this challenge model. It does not perfectly reflect natural infection because P. falciparum antigens are expressed under the control of the P. berghei UIS4 promoter and thus expressed on sporozoites and at high levels during liver-stage infection, overall increasing both the duration and level of P. falciparum antigen expression compared to natural expression in P. falciparum. In the absence of P. berghei orthologues for PfLSA1 and PfLSAP2, only expression of these antigens as an additional gene and not gene replacement parasite could be tested. PfLSAP2 is still a relatively new liver-stage antigen and thus has yet to be tested for immunogenicity and efficacy in human clinical trials. In contrast, PfLSA1 has been assessed in a number of clinical trials as a protein vaccine (44), in mixed administration of DNA vaccines (45), or as a component of an epitope string expressed in poxvirus vectors (46, 47). Still, the level of T cell-mediated immunogenicity against PfLSA1 in all studies was low, which may explain the lack of efficacy observed.

Given that the expression of both PfLSA1 and PfLSAP2 is associated with the PVM, it is more likely that if these antigens are to be efficacious in humans, induction of cell-mediated immunity rather than induction of antibodies would be a more plausible mechanism of protection. With viral vectors now capable of inducing high numbers of circulating T cells in humans (12) and two antigens that demonstrate T cell-mediated protection in preclinical models (18), both PfLSA1 and PfLSAP2 make attractive candidate antigens to test in humans. As inclusion of multiple antigens in a vaccine increases the likelihood of reaching the necessary number and antigenic breadth of T cells required for protection, by broadening the antigen repertoire and potentially increasing the total number of antigen-specific cells, combining the two antigens may provide the highly efficacious vaccine required to control malaria.

In this study, we have demonstrated that inclusion of two P. falciparum liver-stage antigens in viral vectors does not significantly impact immunogenicity or efficacy when tested in a chimeric sporozoite challenge model at the peak of the T cell response. As the only way to truly test the efficacy of PfLSA1 and PfLSAP2 antigens is in a controlled human malaria infection (CHMI) study, the adenoviral vaccines ChAdOx1-sharkTM/Ii-LSA1-LSAP2 and MVA-tPA-LSA1-LSAP2 have been produced to clinical grade (under GMP guidelines) and will undergo assessment of immunogenicity and efficacy in human clinical trials.

## MATERIALS AND METHODS

### Construction of recombinant adenovirus vectors.

PfLSA1- and PfLSAP2-expressing constructs (18) were subcloned into a transgene expression cassette comprising a modified human cytomegalovirus major immediate-early promoter (CMV promoter) with tetracycline operator (TetO) sites. The cassettes were inserted into the E1 locus of a genomic clone of ChAd63 or ChAdOx1 with E1/E3 deleted and E4 modified using site-specific recombination with the viruses rescued and propagated in T-REx-293 cells and purified by CsCl gradient ultracentrifugation, and titers were determined as previously described. Doses for vaccination were based on infectious units and not viral particles, as infectivity, rather than the viral particle number, is correlated with immunogenicity (24). ChAd63 or ChAdOx1 particle-to-infectious unit (P/I) ratios were in the range of 50 to 120.

### Construction of recombinant MVA.

PfLSA1 and PfLSAP2 constructs (18) were subcloned into an orthopoxviral shuttle plasmid under the control of the vaccinia virus p7.5 promoter. The cassette was introduced into the thymidine kinase (TK) locus of MVA by recombination in transfected and infected chicken embryo fibroblasts (CEF), followed by transient selection with a green fluorescent protein (GFP) marker gene. The resulting markerless viral recombinants were plaque purified, amplified in CEF, and titrated using an immunostaining plaque assay according to standard methods. The identity and purity of the isolates were verified by PCR. Doses for vaccination were based on plaque-forming units (PFU).

### Ethics statement.

All animal work was conducted in accordance with the U.K. Animals (Scientific Procedures) Act of 1986 and approved by the University of Oxford Animal Care and Ethical Review Committee for use under project licenses 30/2889 and P9804B4F1. All experimental animal work conducted at the Leiden University Medical Center (LUMC) (Leiden, The Netherlands) was approved by the Animal Experiments Committee of the LUMC (DEC 12042). Animals were group housed in individually ventilated cages under specific-pathogen-free conditions with constant temperature and humidity and with a 12:12 light-dark cycle (8 a.m. to 8 p.m.). For induction of short-term anesthesia, animals were either injected intramuscularly (i.m.) with xylazine and ketamine or anesthetized using vaporized IsoFlo (Zoetis). All the animals were humanely sacrificed at the end of each experiment by an approved schedule 1 method. All efforts were made to minimize suffering.

### Animals and immunizations.

Female BALB/cOlaHsd (BALB/c) or CD-1 (ICR) mice at least 6 weeks of age (Envigo) were given intramuscular (i.m.) immunizations into the musculus tibialis with a total volume of 50 μl of vaccine diluted in endotoxin-free phosphate-buffered saline (PBS) using a 29-gauge 0.5-ml insulin syringe (BD). When two vaccines were coadministered, full doses were delivered individually into separate legs or mixed and delivered into both legs (2 doses of 50 μl each).

Chimeric P. berghei parasites expressing the P. falciparum antigen of interest were generated by “gene insertion/marker out” (GIMO) technology as described below. To enable selection of transfected parasites, mice were injected with blood-stage parasites, and pyrimethamine was added to the drinking water.

### Antigens for *in vitro* restimulation.

Peptides used in immunological assays were purchased from commercial suppliers (NeoScientific, Woburn, MA, USA; Mimotopes, Wirral, United Kingdom; or Thermo Fisher Scientific). Peptides overlapping by 10 aa for the entire protein sequence of P. falciparum TRAP (greater than 80% purity) (48), P. falciparum LSA1 (crude), or P. falciparum LSAP2 (crude) (49) were used. The peptides were reconstituted in dimethyl sulfoxide (DMSO) at 50 to 100 mg/ml depending on their solubilities and combined into a final peptide pool for cellular assays with DMSO at a final concentration of less than 1%.

### Intracellular cytokine staining (ICS).

Peripheral blood mononuclear cells (PBMCs) or splenocytes were plated in 96-well round-bottom plates and stimulated by the addition of pools of overlapping 20-mers covering the whole protein at a final concentration of 2 μg/ml in the presence of 1 μg/ml BD GolgiPlug and incubated for 6 h (37°C; 5% CO_2_). After cell surface labeling with anti-CD4-e450 and anti-CD8-peridinin chlorophyll protein (PerCP)/Cy5.5 antibodies (Affymetrix eBioscience), as well as a LIVE/DEAD fixable aqua dead cell stain kit (Thermo Fisher Scientific), the cells were fixed with neutral buffered formalin solution containing 4% formaldehyde (Sigma-Aldrich) for 5 min at 4°C. Then, intracellular staining was performed with anti-tumor necrosis factor (TNF)-Alexa 488, anti-interleukin 2 (IL-2)-phycoerythrin (PE), and anti-IFN-γ–Alexa 647 antibodies (Affymetrix eBioscience) diluted in BD Perm/Wash buffer. Flow cytometry data were analyzed using a BD LSR II flow cytometer with BD FACSDIVA (Becton, Dickinson) and FlowJo (Tree Star) software. Antigen-specific cells were identified by gating on size, double-negative live cells, and either CD4^+^ or CD8^+^ surface expression. Background responses in unstimulated wells were subtracted from responses of stimulated T cells before statistical analysis in Prism 6.07 (GraphPad) (see Fig. S7 in the supplemental material).

### Parasites and efficacy studies.

Sporozoites were obtained by dissection and homogenization of salivary glands from Anopheles stephensi mosquitoes 21 days postinfection. The generation of chimeric P. berghei-P. falciparum parasites expressing PfLSA1 has been described previously (18). In addition, we generated double-chimeric P. berghei-P. falciparum parasites that express both PfTRAP and PfLSA1, PfTRAP and PfLSAP2, or PfLSA1 and PfLSAP2 under the control of the *Pbuis4* promoter (see below). For challenge experiments, 1,000 sporozoites were injected intravenously (i.v.) into the tail vein 8 to 10 days following the final vaccination, and mice were monitored from 4 days postinfection by Giemsa-stained thin-film blood smears. The experimental endpoint was 14 or 15 days parasite free (sterilely protected) or when blood-stage parasites had been confirmed on three consecutive days. The time to 1% parasitemia was calculated using linear regression; when sterile protection was not achieved, the value was adept at providing a sensitive measure of liver-stage protection, as it reflects numbers of parasites erupting from the liver (50, 51).

### Generation of P. berghei-P. falciparum double-chimeric parasites.

Double-additional-gene (DAG) chimeric parasites were generated as previously described (38) by using a single additional gene (SAG) as the background parent line and stably inserting the additional P. falciparum gene into the neutral *s1* gene locus in chromosome 12 through double-crossover recombination using a 2-step GIMO transfection protocol (52, 53) (see Fig. S8 in the supplemental material).

In the first step, we deleted the Pbs1 coding DNA sequence (CDS) and replaced it with a positive-negative selectable marker to create a *Pbs1* deletion GIMO line from the parent line. In order to do this, we generated the plasmid construct based on the standard GIMO DNA construct pL0034, which contains a positive-negative (h*dhfr*::y*fcu*) SM cassette and was used to insert both the *Pbs1* 5′ and 3′ gene-targeting regions (TRs). The linear pL1928 DNA construct was introduced into the parent plasmid by using standard methods of transfection. The transfected parasites were selected in mice through addition of pyrimethamine in the drinking water. The transfected parasites were cloned by limiting dilution, resulting in the PbANKA-P. falciparum Ag plus PbΔs1 GIMO line.

Correct integration of the constructs into the genomes of chimeric parasites was analyzed by diagnostic PCR analysis of genomic DNA (gDNA) and Southern analysis of pulsed-field gel electrophoresis-separated chromosomes (see Fig. S9 in the supplemental material). The primers used for PCR genotyping are listed in Table S1 in the supplemental material.

Antigen expression by chimeric parasites was confirmed by immunofluorescence assay (IFA) staining of sporozoites with sera from mice vaccinated with a single antigen, PfLSA1, PfLSAP2, or PfTRAP (see Fig. S10 in the supplemental material).

### Statistical analysis.

The statistical software Prism version 6 (GraphPad) was used for all analyses. Survival in challenge experiments is presented using Kaplan-Meier curves with significance tested using the log rank (Mantel-Cox) test. For immunogenicity analysis, the data in each graph were analyzed with one-way analysis of variance (ANOVA) and *post hoc* Kruskal-Wallace tests. Nonparametric data are shown as medians with individual data points plotted unless otherwise indicated. Each figure represents an independent *in vivo* experiment. Because of the variability between *in vivo* experiments due to vaccine preparations, batches of sporozoites, or *ex vivo* stimulation performed on different days, replicate experiments were not pooled, and representative experiments are shown.

## Supplementary Material

Supplemental file 1

## References

[1] WHO. 2015 World Malaria Report 2015. WHO, Geneva, Switzerland.

[2] RTS,S Clinical Trials Partnership. 2015 Efficacy, and safety of RTS,S/AS01 malaria vaccine with or without a booster dose in infants and children in Africa: final results of a phase 3, individually randomised, controlled trial. Lancet 386:31–45. doi:10.1016/S0140-6736(15)60721-8. 25913272 PMC5626001

[3] Ewer KJ, O'Hara GA, Duncan CJA, Collins KA, Sheehy SH, Reyes-Sandoval A, Goodman AL, Edwards NJ, Elias SC, Halstead FD, Longley RJ, Rowland R, Poulton ID, Draper SJ, Blagborough AM, Berrie E, Moyle S, Williams N, Siani L, Folgori A, Colloca S, Sinden RE, Lawrie AM, Cortese R, Gilbert SC, Nicosia A, Hill AVS 2013 Protective CD8+ T-cell immunity to human malaria induced by chimpanzee adenovirus-MVA immunisation. Nat Commun 4:2836. doi:10.1038/ncomms3836. 24284865 PMC3868203

[4] Ogwang C, Kimani D, Edwards NJ, Roberts R, Mwacharo J, Bowyer G, Bliss C, Hodgson SH, Njuguna P, Viebig NK, Nicosia A, Gitau E, Douglas S, Illingworth J, Marsh K, Lawrie A, Imoukhuede EB, Ewer K, Urban BC, Hill AVS, Bejon P, MVVC Group 2015 Prime-boost vaccination with chimpanzee adenovirus and modified vaccinia Ankara encoding TRAP provides partial protection against Plasmodium falciparum infection in Kenyan adults. Sci Transl Med 7:286re285. doi:10.1126/scitranslmed.aaa2373. PMC4687051 25947165

[5] Chuang I, Sedegah M, Cicatelli S, Spring M, Polhemus M, Tamminga C, Patterson N, Guerrero M, Bennett JW, McGrath S, Ganeshan H, Belmonte M, Farooq F, Abot E, Banania JG, Huang J, Newcomer R, Rein L, Litilit D, Richie NO, Wood C, Murphy J, Sauerwein R, Hermsen CC, McCoy AJ, Kamau E, Cummings J, Komisar J, Sutamihardja A, Shi M, Epstein JE, Maiolatesi S, Tosh D, Limbach K, Angov E, Bergmann-Leitner E, Bruder JT, Doolan DL, King CR, Carucci D, Dutta S, Soisson L, Diggs C, Hollingdale MR, Ockenhouse CF, Richie TL 2013 DNA prime/adenovirus boost malaria vaccine encoding P falciparum CSP and AMA1 induces sterile protection associated with cell-mediated immunity. PLoS One 8:e55571. doi:10.1371/journal.pone.0055571. 23457473 PMC3573028

[6] Seder RA, Chang LJ, Enama ME, Zephir KL, Sarwar UN, Gordon IJ, Holman LA, James ER, Billingsley PF, Gunasekera A, Richman A, Chakravarty S, Manoj A, Velmurugan S, Li M, Ruben AJ, Li T, Eappen AG, Stafford RE, Plummer SH, Hendel CS, Novik L, Costner PJ, Mendoza FH, Saunders JG, Nason MC, Richardson JH, Murphy J, Davidson SA, Richie TL, Sedegah M, Sutamihardja A, Fahle GA, Lyke KE, Laurens MB, Roederer M, Tewari K, Epstein JE, Sim BK, Ledgerwood JE, Graham BS, Hoffman SL, VRC 312 StudyTeam 2013 Protection against malaria by intravenous immunization with a nonreplicating sporozoite vaccine. Science 341:1359–1365. doi:10.1126/science.1241800. 23929949

[7] Mordmuller B, Surat G, Lagler H, Chakravarty S, Ishizuka AS, Lalremruata A, Gmeiner M, Campo JJ, Esen M, Ruben AJ, Held J, Calle CL, Mengue JB, Gebru T, Ibanez J, Sulyok M, James ER, Billingsley PF, Natasha KC, Manoj A, Murshedkar T, Gunasekera A, Eappen AG, Li T, Stafford RE, Li M, Felgner PL, Seder RA, Richie TL, Sim BK, Hoffman SL, Kremsner PG 2017 Sterile protection against human malaria by chemoattenuated PfSPZ vaccine. Nature 542:445–449. doi:10.1038/nature21060. 28199305 PMC10906480

[8] Romero P, Maryanski JL, Corradin G, Nussenzweig RS, Nussenzweig V, Zavala F 1989 Cloned cytotoxic T cells recognize an epitope in the circumsporozoite protein and protect against malaria. Nature 341:323–326. doi:10.1038/341323a0. 2477703

[9] Schofield L, Villaquiran J, Ferreira A, Schellekens H, Nussenzweig R, Nussenzweig V 1987 Gamma interferon, CD8+ T cells and antibodies required for immunity to malaria sporozoites. Nature 330:664–666. doi:10.1038/330664a0. 3120015

[10] Weiss WR, Sedegah M, Beaudoin RL, Miller LH, Good MF 1988 CD8+ T cells (cytotoxic/suppressors) are required for protection in mice immunized with malaria sporozoites. Proc Natl Acad Sci U S A 85:573–576. doi:10.1073/pnas.85.2.573. 2963334 PMC279593

[11] Spencer AJ, Longley RJ, Gola A, Ulaszewska M, Lambe T, Hill AV 2017 The threshold of protection from liver-stage malaria relies on a fine balance between the number of infected hepatocytes and effector CD8(+) T cells present in the liver. J Immunol 198:2006–2016. doi:10.4049/jimmunol.1601209. 28087668 PMC5318841

[12] Ewer K, Sebastian S, Spencer AJ, Gilbert S, Hill AVS, Lambe T 2017 Chimpanzee adenoviral vectors as vaccines for outbreak pathogens. Hum Vaccin Immunother 13:3020–3032. doi:10.1080/21645515.2017.1383575. 29083948 PMC5718829

[13] O'Hara GA, Duncan CJA, Ewer KJ, Collins KA, Elias SC, Halstead FD, Goodman AL, Edwards NJ, Reyes-Sandoval A, Bird P, Rowland R, Sheehy SH, Poulton ID, Hutchings C, Todryk S, Andrews L, Folgori A, Berrie E, Moyle S, Nicosia A, Colloca S, Cortese R, Siani L, Lawrie AM, Gilbert SC, Hill AVS 2012 Clinical assessment of a recombinant simian adenovirus ChAd63: a potent new vaccine vector. J Infect Dis 205:772–781. doi:10.1093/infdis/jir850. 22275401 PMC3274376

[14] Good MF, Doolan DL 2010 Malaria vaccine design: immunological considerations. Immunity 33:555–566. doi:10.1016/j.immuni.2010.10.005. 21029965

[15] Spencer AJ, Cottingham MG, Jenks JA, Longley RJ, Capone S, Colloca S, Folgori A, Cortese R, Nicosia A, Bregu M, Hill AV 2014 Enhanced vaccine-induced CD8+ T cell responses to malaria antigen ME-TRAP by fusion to MHC class II invariant chain. PLoS One 9:e100538. doi:10.1371/journal.pone.0100538. 24945248 PMC4063960

[16] Halbroth BR, Sebastian S, Poyntz HC, Bregu M, Cottingham MG, Hill AVS, Spencer AJ 2018 Development of a molecular adjuvant to enhance antigen-specific CD8(+) T cell responses. Sci Rep 8:15020. doi:10.1038/s41598-018-33375-1. 30301933 PMC6177389

[17] Duffy PE, Sahu T, Akue A, Milman N, Anderson C 2012 Pre-erythrocytic malaria vaccines: identifying the targets. Expert Rev Vaccines 11:1261–1280. doi:10.1586/erv.12.92. 23176657 PMC3584156

[18] Longley RJ, Salman AM, Cottingham MG, Ewer K, Janse CJ, Khan SM, Spencer AJ, Hill AV 2015 Comparative assessment of vaccine vectors encoding ten malaria antigens identifies two protective liver-stage candidates. Sci Rep 5:11820. doi:10.1038/srep11820. 26139288 PMC4490344

[19] Doolan DL, Sedegah M, Hedstrom RC, Hobart P, Charoenvit Y, Hoffman SL 1996 Circumventing genetic restriction of protection against malaria with multigene DNA immunization: CD8+ cell-, interferon gamma-, and nitric oxide-dependent immunity. J Exp Med 183:1739–1746. doi:10.1084/jem.183.4.1739. 8666931 PMC2192484

[20] Pichyangkul S, Tongtawe P, Kum-Arb U, Yongvanitchit K, Gettayacamin M, Hollingdale MR, Limsalakpetch A, Stewart VA, Lanar DE, Dutta S, Angov E, Ware LA, Bergmann-Leitner ES, House B, Voss G, Dubois MC, Cohen JD, Fukuda MM, Heppner DG, Miller RS 2009 Evaluation of the safety and immunogenicity of Plasmodium falciparum apical membrane antigen 1, merozoite surface protein 1 or RTS,S vaccines with adjuvant system AS02A administered alone or concurrently in rhesus monkeys. Vaccine 28:452–462. doi:10.1016/j.vaccine.2009.10.022. 19857448

[21] Grifantini R, Finco O, Bartolini E, Draghi M, Del Giudice G, Kocken C, Thomas A, Abrignani S, Grandi G 1998 Multi-plasmid DNA vaccination avoids antigenic competition and enhances immunogenicity of a poorly immunogenic plasmid. Eur J Immunol 28:1225–1232. doi:10.1002/(SICI)1521-4141(199804)28:04<1225::AID-IMMU1225>3.0.CO;2-6. 9565362

[22] Reyes-Sandoval A, Sridhar S, Berthoud T, Moore AC, Harty JT, Gilbert SC, Gao G, Ertl HC, Wilson JC, Hill AV 2008 Single-dose immunogenicity and protective efficacy of simian adenoviral vectors against Plasmodium berghei. Eur J Immunol 38:732–741. doi:10.1002/eji.200737672. 18266272

[23] Reyes-Sandoval A, Berthoud T, Alder N, Siani L, Gilbert SC, Nicosia A, Colloca S, Cortese R, Hill AV 2010 Prime-boost immunization with adenoviral and modified vaccinia virus Ankara vectors enhances the durability and polyfunctionality of protective malaria CD8+ T-cell responses. Infect Immun 78:145–153. doi:10.1128/IAI.00740-09. 19858306 PMC2798185

[24] Dicks MD, Spencer AJ, Edwards NJ, Wadell G, Bojang K, Gilbert SC, Hill AV, Cottingham MG 2012 A novel chimpanzee adenovirus vector with low human seroprevalence: improved systems for vector derivation and comparative immunogenicity. PLoS One 7:e40385. doi:10.1371/journal.pone.0040385. 22808149 PMC3396660

[25] Gola A, Silman D, Walters AA, Sridhar S, Uderhardt S, Salman AM, Halbroth BR, Bellamy D, Bowyer G, Powlson J, Baker M, Venkatraman N, Poulton I, Berrie E, Roberts R, Lawrie AM, Angus B, Khan SM, Janse CJ, Ewer KJ, Germain RN, Spencer AJ, Hill A 2018 Prime and target immunization protects against liver-stage malaria in mice. Sci Transl Med 10:eaap9128. doi:10.1126/scitranslmed.aap9128. 30257955

[26] Doolan DL, Southwood S, Freilich DA, Sidney J, Graber NL, Shatney L, Bebris L, Florens L, Dobano C, Witney AA, Appella E, Hoffman SL, Yates JR III, Carucci DJ, Sette A 2003 Identification of Plasmodium falciparum antigens by antigenic analysis of genomic and proteomic data. Proc Natl Acad Sci U S A 100:9952–9957. doi:10.1073/pnas.1633254100. 12886016 PMC187898

[27] Gruner AC, Mauduit M, Tewari R, Romero JF, Depinay N, Kayibanda M, Lallemand E, Chavatte JM, Crisanti A, Sinnis P, Mazier D, Corradin G, Snounou G, Renia L 2007 Sterile protection against malaria is independent of immune responses to the circumsporozoite protein. PLoS One 2:e1371. doi:10.1371/journal.pone.0001371. 18159254 PMC2147056

[28] Kumar KA, Sano G, Boscardin S, Nussenzweig RS, Nussenzweig MC, Zavala F, Nussenzweig V 2006 The circumsporozoite protein is an immunodominant protective antigen in irradiated sporozoites. Nature 444:937–940. doi:10.1038/nature05361. 17151604

[29] Mauduit M, Tewari R, Depinay N, Kayibanda M, Lallemand E, Chavatte JM, Snounou G, Renia L, Gruner AC 2010 Minimal role for the circumsporozoite protein in the induction of sterile immunity by vaccination with live rodent malaria sporozoites. Infect Immun 78:2182–2188. doi:10.1128/IAI.01415-09. 20194600 PMC2863544

[30] Siau A, Silvie O, Franetich JF, Yalaoui S, Marinach C, Hannoun L, van Gemert GJ, Luty AJ, Bischoff E, David PH, Snounou G, Vaquero C, Froissard P, Mazier D 2008 Temperature shift and host cell contact up-regulate sporozoite expression of Plasmodium falciparum genes involved in hepatocyte infection. PLoS Pathog 4:e1000121. doi:10.1371/journal.ppat.1000121. 18688281 PMC2488394

[31] Fidock DA, Gras-Masse H, Lepers JP, Brahimi K, Benmohamed L, Mellouk S, Guerin-Marchand C, Londono A, Raharimalala L, Meis JF 1994 Plasmodium falciparum liver stage antigen-1 is well conserved and contains potent B and T cell determinants. J Immunol 153:190–204. 7515922

[32] Mikolajczak SA, Sacci JB Jr, De La Vega P, Camargo N, VanBuskirk K, Krzych U, Cao J, Jacobs-Lorena M, Cowman AF, Kappe SH 2011 Disruption of the Plasmodium falciparum liver-stage antigen-1 locus causes a differentiation defect in late liver-stage parasites. Cell Microbiol 13:1250–1260. doi:10.1111/j.1462-5822.2011.01617.x. 21569184 PMC4155577

[33] John CC, Sumba PO, Ouma JH, Nahlen BL, King CL, Kazura JW 2000 Cytokine responses to Plasmodium falciparum liver-stage antigen 1 vary in rainy and dry seasons in highland Kenya. Infect Immun 68:5198–5204. doi:10.1128/iai.68.9.5198-5204.2000. 10948144 PMC101778

[34] Kurtis JD, Lanar DE, Opollo M, Duffy PE 1999 Interleukin-10 responses to liver-stage antigen 1 predict human resistance to Plasmodium falciparum. Infect Immun 67:3424–3429. 10377122 10.1128/iai.67.7.3424-3429.1999 PMC116527

[35] Luty AJ, Lell B, Schmidt-Ott R, Lehman LG, Luckner D, Greve B, Matousek P, Herbich K, Schmid D, Ulbert S, Migot-Nabias F, Dubois B, Deloron P, Kremsner PG 1998 Parasite antigen-specific interleukin-10 and antibody responses predict accelerated parasite clearance in Plasmodium falciparum malaria. Eur Cytokine Netw 9:639–646. 9889408

[36] Migot-Nabias F, Deloron P, Ringwald P, Dubois B, Mayombo J, Minh TN, Fievet N, Millet P, Luty A 2000 Immune response to Plasmodium falciparum liver stage antigen-1: geographical variations within Central Africa and their relationship with protection from clinical malaria. Trans R Soc Trop Med Hyg 94:557–562. doi:10.1016/s0035-9203(00)90086-5. 11132389

[37] Krzych U, Lyon JA, Jareed T, Schneider I, Hollingdale MR, Gordon DM, Ballou WR 1995 T lymphocytes from volunteers immunized with irradiated Plasmodium falciparum sporozoites recognize liver and blood stage malaria antigens. J Immunol 155:4072–4077. 7561118

[38] Longley RJ, Halbroth BR, Salman AM, Ewer KJ, Hodgson SH, Janse CJ, Khan SM, Hill AV, Spencer AJ 2017 Assessment of the Plasmodium falciparum preerythrocytic antigen UIS3 as a potential candidate for a malaria vaccine. Infect Immun 85:e00641-16. doi:10.1128/IAI.00641-16. PMC5328496 28031267

[39] Mould RC, AuYeung AWK, van Vloten JP, Susta L, Mutsaers AJ, Petrik JJ, Wood GA, Wootton SK, Karimi K, Bridle BW 2017 Enhancing immune responses to cancer vaccines using multi-site injections. Sci Rep 7:8322. doi:10.1038/s41598-017-08665-9. 28814733 PMC5559552

[40] Humphreys IR, Sebastian S 2018 Novel viral vectors in infectious diseases. Immunology 153:1–9. doi:10.1111/imm.12829. 28869761 PMC5721250

[41] Luo M, Tao P, Li J, Zhou S, Guo D, Pan Z 2008 Immunization with plasmid DNA encoding influenza A virus nucleoprotein fused to a tissue plasminogen activator signal sequence elicits strong immune responses and protection against H5N1 challenge in mice. J Virol Methods 154:121–127. doi:10.1016/j.jviromet.2008.08.011. 18789973

[42] Becker SI, Wang R, Hedstrom RC, Aguiar JC, Jones TR, Hoffman SL, Gardner MJ 1998 Protection of mice against Plasmodium yoelii sporozoite challenge with P. yoelii merozoite surface protein 1 DNA vaccines. Infect Immun 66:3457–3461. 9632624 10.1128/iai.66.7.3457-3461.1998 PMC108371

[43] Li Z, Howard A, Kelley C, Delogu G, Collins F, Morris S 1999 Immunogenicity of DNA vaccines expressing tuberculosis proteins fused to tissue plasminogen activator signal sequences. Infect Immun 67:4780–4786. 10456931 10.1128/iai.67.9.4780-4786.1999 PMC96809

[44] Cummings JF, Spring MD, Schwenk RJ, Ockenhouse CF, Kester KE, Polhemus ME, Walsh DS, Yoon IK, Prosperi C, Juompan LY, Lanar DE, Krzych U, Hall BT, Ware LA, Stewart VA, Williams J, Dowler M, Nielsen RK, Hillier CJ, Giersing BK, Dubovsky F, Malkin E, Tucker K, Dubois MC, Cohen JD, Ballou WR, Heppner DG Jr. 2010 Recombinant liver stage antigen-1 (LSA-1) formulated with AS01 or AS02 is safe, elicits high titer antibody and induces IFN-gamma/IL-2 CD4+ T cells but does not protect against experimental Plasmodium falciparum infection. Vaccine 28:5135–5144. doi:10.1016/j.vaccine.2009.08.046. 19737527

[45] Richie TL, Charoenvit Y, Wang R, Epstein JE, Hedstrom RC, Kumar S, Luke TC, Freilich DA, Aguiar JC, Sacci JB Jr, Sedegah M, Nosek RA Jr, De La Vega P, Berzins MP, Majam VF, Abot EN, Ganeshan H, Richie NO, Banania JG, Baraceros MF, Geter TG, Mere R, Bebris L, Limbach K, Hickey BW, Lanar DE, Ng J, Shi M, Hobart PM, Norman JA, Soisson LA, Hollingdale MR, Rogers WO, Doolan DL, Hoffman SL 2012 Clinical trial in healthy malaria-naive adults to evaluate the safety, tolerability, immunogenicity and efficacy of MuStDO5, a five-gene, sporozoite/hepatic stage Plasmodium falciparum DNA vaccine combined with escalating dose human GM-CSF DNA. Hum Vaccin Immunother 8:1564–1584. doi:10.4161/hv.22129. 23151451 PMC3601132

[46] Porter DW, Thompson FM, Berthoud TK, Hutchings CL, Andrews L, Biswas S, Poulton I, Prieur E, Correa S, Rowland R, Lang T, Williams J, Gilbert SC, Sinden RE, Todryk S, Hill AV 2011 A human phase I/IIa malaria challenge trial of a polyprotein malaria vaccine. Vaccine 29:7514–7522. doi:10.1016/j.vaccine.2011.03.083. 21501642 PMC3195259

[47] Ockenhouse CF, Sun PF, Lanar DE, Wellde BT, Hall BT, Kester K, Stoute JA, Magill A, Krzych U, Farley L, Wirtz RA, Sadoff JC, Kaslow DC, Kumar S, Church LW, Crutcher JM, Wizel B, Hoffman S, Lalvani A, Hill AV, Tine JA, Guito KP, de Taisne C, Anders R, Ballou WR 1998 Phase I/IIa safety, immunogenicity, and efficacy trial of NYVAC-Pf7, a pox-vectored, multiantigen, multistage vaccine candidate for Plasmodium falciparum malaria. J Infect Dis 177:1664–1673. doi:10.1086/515331. 9607847

[48] McConkey SJ, Reece WH, Moorthy VS, Webster D, Dunachie S, Butcher G, Vuola JM, Blanchard TJ, Gothard P, Watkins K, Hannan CM, Everaere S, Brown K, Kester KE, Cummings J, Williams J, Heppner DG, Pathan A, Flanagan K, Arulanantham N, Roberts MT, Roy M, Smith GL, Schneider J, Peto T, Sinden RE, Gilbert SC, Hill AV 2003 Enhanced T-cell immunogenicity of plasmid DNA vaccines boosted by recombinant modified vaccinia virus Ankara in humans. Nat Med 9:729–735. doi:10.1038/nm881. 12766765

[49] Longley RJ, Halbroth BR, Ewer KJ, Hill AV, Spencer AJ 2015 Identification of immunodominant responses to the Plasmodium falciparum antigens PfUIS3, PfLSA1 and PfLSAP2 in multiple strains of mice. PLoS One 10:e0144515. doi:10.1371/journal.pone.0144515. 26659715 PMC4676683

[50] Hafalla JC, Bauza K, Friesen J, Gonzalez-Aseguinolaza G, Hill AV, Matuschewski K 2013 Identification of targets of CD8(+) T cell responses to malaria liver stages by genome-wide epitope profiling. PLoS Pathog 9:e1003303. doi:10.1371/journal.ppat.1003303. 23675294 PMC3649980

[51] Bejon P, Andrews L, Andersen RF, Dunachie S, Webster D, Walther M, Gilbert SC, Peto T, Hill AV 2005 Calculation of liver-to-blood inocula, parasite growth rates, and preerythrocytic vaccine efficacy, from serial quantitative polymerase chain reaction studies of volunteers challenged with malaria sporozoites. J Infect Dis 191:619–626. doi:10.1086/427243. 15655787

[52] Salman AM, Mogollon CM, Lin JW, van Pul FJ, Janse CJ, Khan SM 2015 Generation of transgenic rodent malaria parasites expressing human malaria parasite proteins. Methods Mol Biol 1325:257–286. doi:10.1007/978-1-4939-2815-6_21. 26450395

[53] Lin JW, Annoura T, Sajid M, Chevalley-Maurel S, Ramesar J, Klop O, Franke-Fayard BM, Janse CJ, Khan SM 2011 A novel ‘gene insertion/marker out’ (GIMO) method for transgene expression and gene complementation in rodent malaria parasites. PLoS One 6:e29289. doi:10.1371/journal.pone.0029289. 22216235 PMC3246482

