# Supplementary material for: Preclinical Development and Assessment of Viral Vectors Expressing a Fusion Antigen of Plasmodium falciparum LSA1 and LSAP2 for Efficacy against Liver-Stage Malaria
Source: Infect Immun. 2020 Jan 22;88(2):e00573-19. doi: 10.1128/IAI.00573-19 (PMC6977128; doi:10.1128/IAI.00573-19)
Supplement: Supplemental file 1 [file IAI.00573-19-s0001.pdf]

Supplementary Information:

Pre-clinical development and assessment of viral vectors expressing a fusion antigen of *P. falciparum* LSA1 and LSAP2 for efficacy against liver-stage malaria

Benedict R Halbroth[1], Sarah Sebastian[1], Ahmed M Salman[1,2], Marta Ulaszewska[1], Anita Gola[1], Rhea J Longley[1,3], Chris J Janse[2], Shahid M Khan[2], Adrian VS Hill[1], Alexandra J Spencer[1]

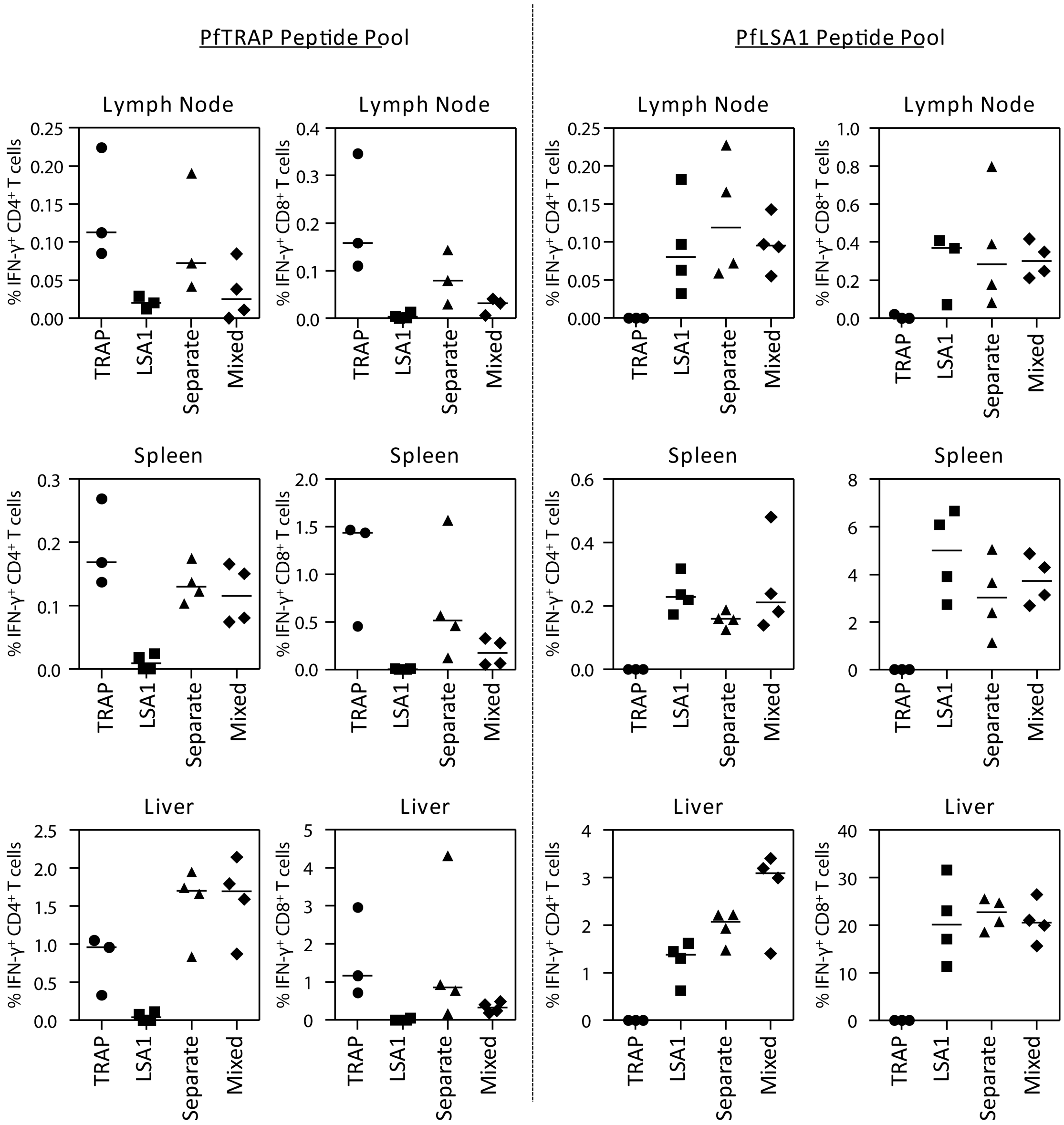

Fig. S1: T-cell responses in lymph nodes, spleen, and liver after vaccine co-administration

BALB/c mice (n=4) were vaccinated with a ChAd63-MVA prime-boost regimen, when two vaccines were given, mice were vaccinated with a full dose of each vaccine administered in separate legs ("Separate") or mixed prior to administration in both legs ("Mixed"). One week post MVA boost (week 7), mice were sacrificed and the popliteal lymph node, spleen, and liver were processed and analysed by ICS after stimulation with a *Pf*TRAP or *Pf*LSA1 peptide pool. Graphs represent the frequency of $CD4^{+}IFN\text{-}\gamma^{+}$ or $CD8^{+}IFN\text{-}\gamma^{+}$ T cells. Single points indicate T-cell responses of individual mice and black lines show the median response per group.

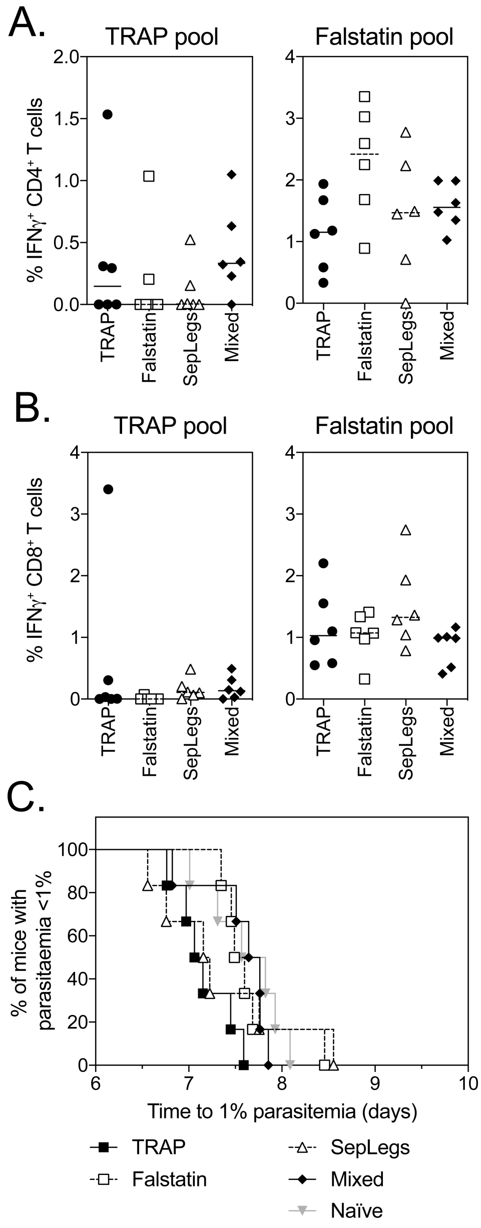

Fig. S2: Immunogenicity and Efficacy of co-administration of PfTRAP and PfFalstatin

BALB/c mice (6 per group) were vaccinated with a 10^8 IU ChAd63 followed 7-weeks later with 10^6 MVA boost of each vaccine expressing PfTRAP (circles), PfFalstatin (squares), both vaccines administered in separate legs (open triangles) or both vaccines mixed together (closed triangles). 1-week post MVA boost (week 7), a blood sample was taken and PBMC were analysed by ICS after stimulation with a PfTRAP or PfFalstatin peptide pool. 10-days after MVA boost mice were challenged with 1000 chimeric *P.berghei* sporozoites expressing *P.falciparum* PfTRAP and PfFalstatin and monitored for development of blood-stage malaria. Graphs represent the frequency of blood $CD4^{+}IFN\text{-}\gamma^{+}$ (**A.**) or $CD8^{+}IFN\text{-}\gamma^{+}$ T cells (**B.**). Single points indicate individual mice, lines show the median response per group. Time to reach 1% parasitaemia is plotted on a Kaplan-Meier Survival Curve (**C.**).

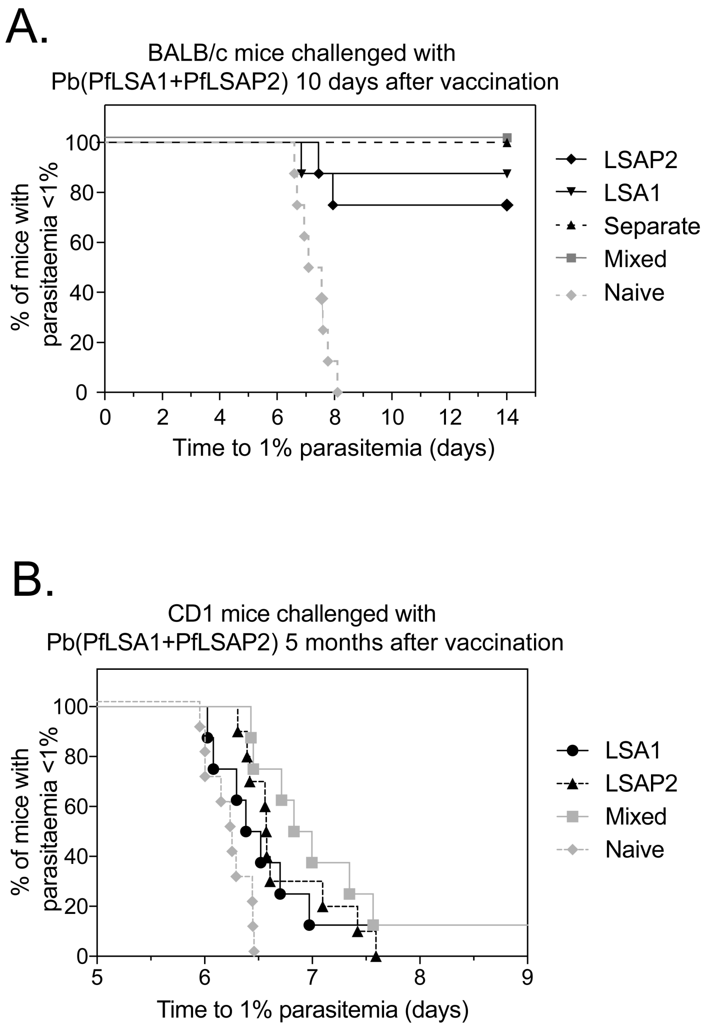

Fig. S3: Efficacy of PfLSA1 and PfLSAP2 combination

A.) BALB/c mice (8 per group) were vaccinated with 10^8 IU ChAd63 and 10^7 PFU MVA encoding either *Pf*LSA1, *Pf*LSAP2 or both in combination. When two vaccines were given, mice were vaccinated with a full dose of each vaccine administered in separate legs ("Separate") or mixed prior to administration in both legs ("Mixed"). Mice were challenged with 1 000 chimeric *P. berghei* sporozoites expressing *P. falciparum* LSA1 and LSAP2 on day 10 post boost monitored for the development of blood stage malaria. Time to 1% parasitaemia is plotted on a Kaplan-Meier Survival Curve.

B.) CD-1 mice (10 per group) were vaccinated with 10^8 IU ChAd63 and 10^7 PFU MVA encoding either *Pf*LSA1, *Pf*LSAP2 or both in combination. Mice were challenged with 1 000 chimeric *P. berghei* sporozoites expressing *P. falciparum* LSA1 and LSAP2 5 months after the MVA boost and monitored for the development of blood stage malaria. Time to 1% parasitaemia is plotted on a Kaplan-Meier Survival Curve.

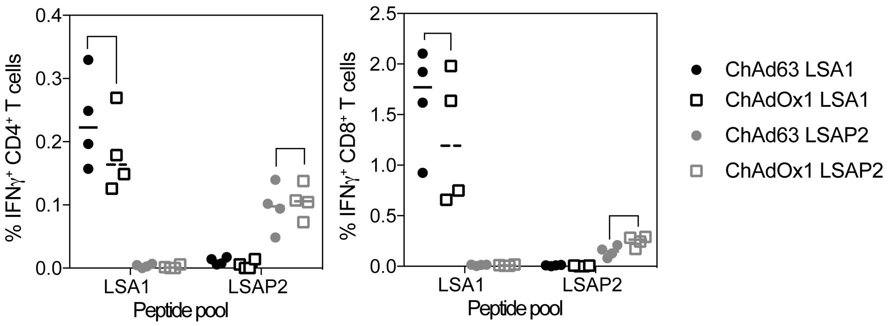

Fig. S4: Immunogenicity of ChAd63 and ChAdOx1

BALB/c mice (4 per group) were immunised with 10^8 IU ChAd63 or ChAdOX1 vectors and spleens harvested two weeks later. T-cell responses to a *Pf*LSA1 or *Pf*LSAP2 peptide pool were analysed by ICS. The percentage of $CD4^+$ and $CD8^+$ T cells positive for IFN-γ are shown. No significant difference between vectors was observed.

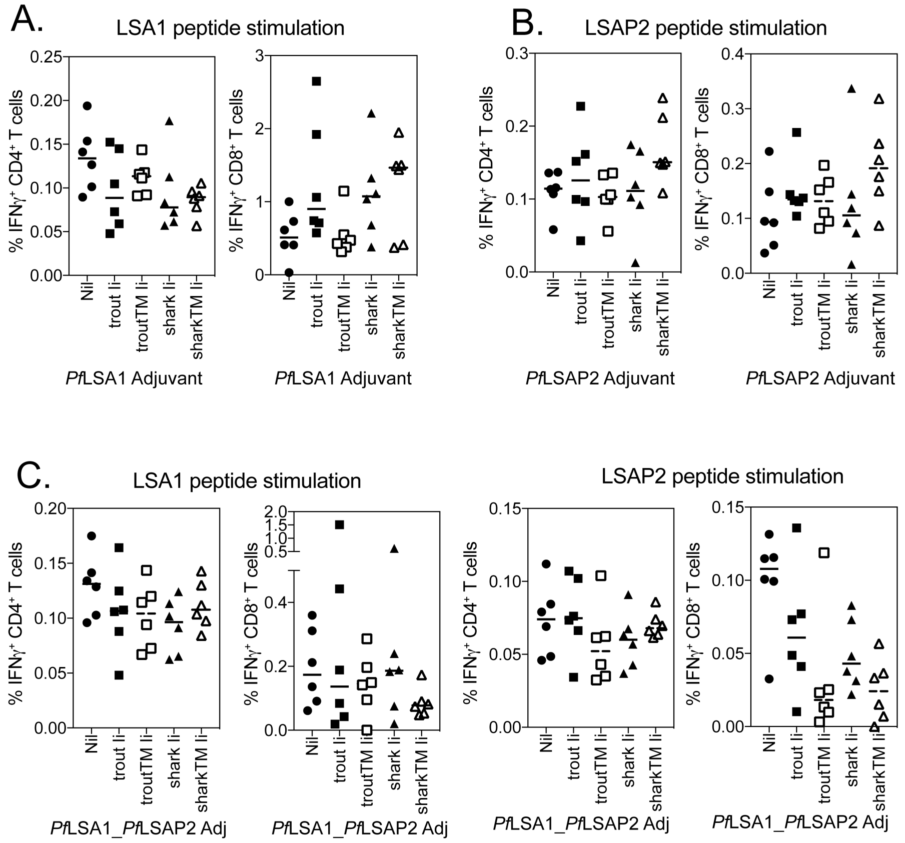

Fig. S5: Effect of molecular antigens on single and dual expressing vectors

BALB/c mice (6 per group) were immunised with 10^7 IU ChAdOX1 vectors expressing *Pf*LSA1 Adjuvant (**A.**), *Pf*LSAP2 Adjuvant (**B.**) or *Pf*LSA1 and *Pf*LSAP2 Adjuvant (**C.**) and spleens were harvested two weeks later. T-cell responses to a *Pf*LSA1 or *Pf*LSAP2 peptide pool were analysed by ICS. The percentage of $CD4^+$ and $CD8^+$ T cells positive for IFN-γ are shown.

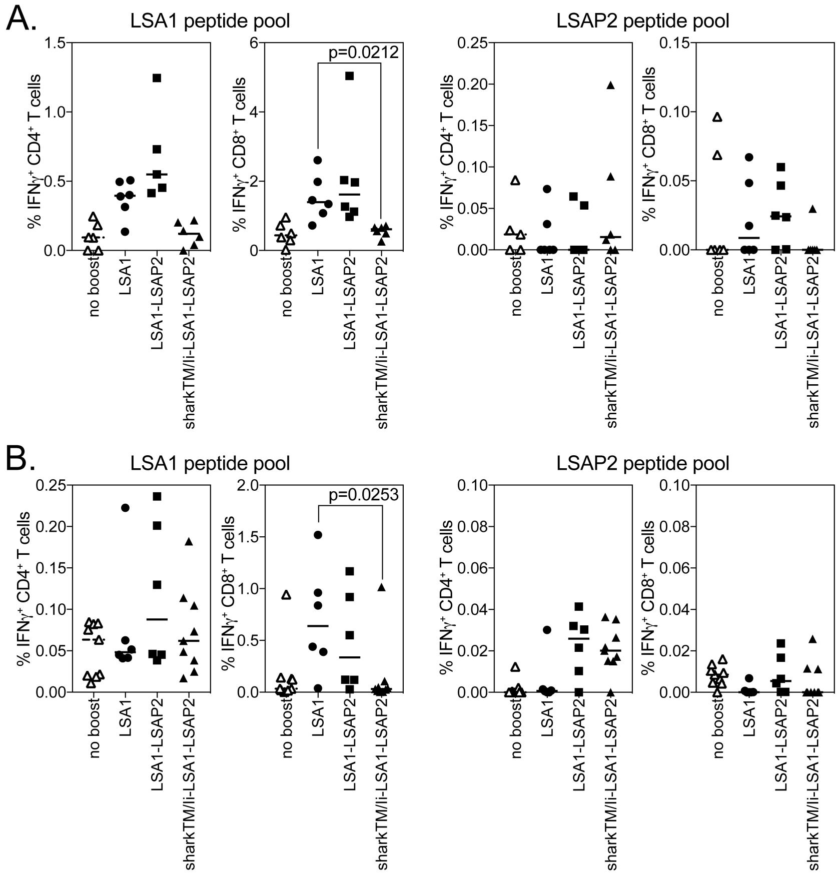

Fig. S6: Immunogenicity of MVA vectors

A.) BALB/c mice (6 per group) were vaccinated with 10^8 IU of ChAdOX1 encoding sharkTM/Ii-LSA1 im followed 1 week later by 10^6 PFU MVA vectors as indicated on the x-axes. A blood sample was taken on day 14 and PBMCs analysed by ICS after stimulation with an LSA1 or LSAP2 peptide pool. Graphs represent the frequency of $CD4^+IFN\text{-}\gamma^+$ or $CD8^+IFN\text{-}\gamma^+$ T cells.

B.) CD1 mice were immunised with 10^8 IU ChAdOX1-sharkTM/Ii-LSA1 and boosted one week later with 10^6 PFU MVA vectors as indicated on the x-axes. Spleens were harvested at day 14. T-cell responses to an LSA1 or LSAP2 peptide pool were analysed by ICS. Graphs represent the frequency of $CD4^+IFN\text{-}\gamma^+$ or $CD8^+IFN\text{-}\gamma^+$ T cells.

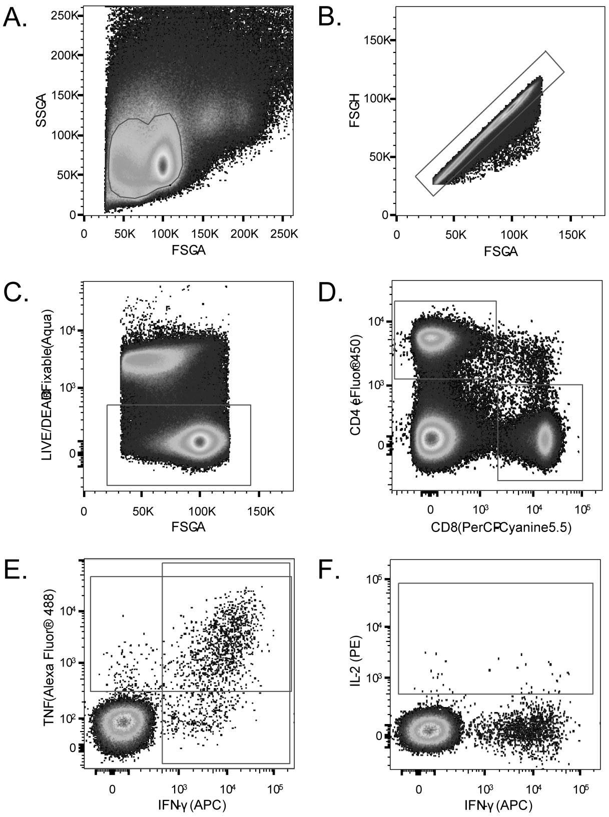

Fig. S7: Flow cytometry gating strategy.

The initial gate was set on the lymphocyte population by analysing the forward scatter (FSC) and side scatter (SSC) data (A), followed by a gate to exclude cell doublets (B). After excluding dead cells (C), lymphocytes were separated into $CD4^+$ or $CD8^+$ T-cell subsets. Finally, cytokine positive cells were gated from those subsets (E and F).

Fig. S8: Double antigen chimeric parasite generation

Double Additional Gene (DAGs) chimeric parasites were generated by using a single additional gene (SAG) as the background parent line and stably inserting the additional *P.falciparum* gene into the neutral *s1* gene locus in chromosome 12 through double-crossover recombination using a 2-step "gene insertion/marker out" (GIMO) transfection protocol. Step 1 involved the replacement of the *s1* gene with a selectable marker, while step 2 enabled the insertion of the additional PfAg gene.

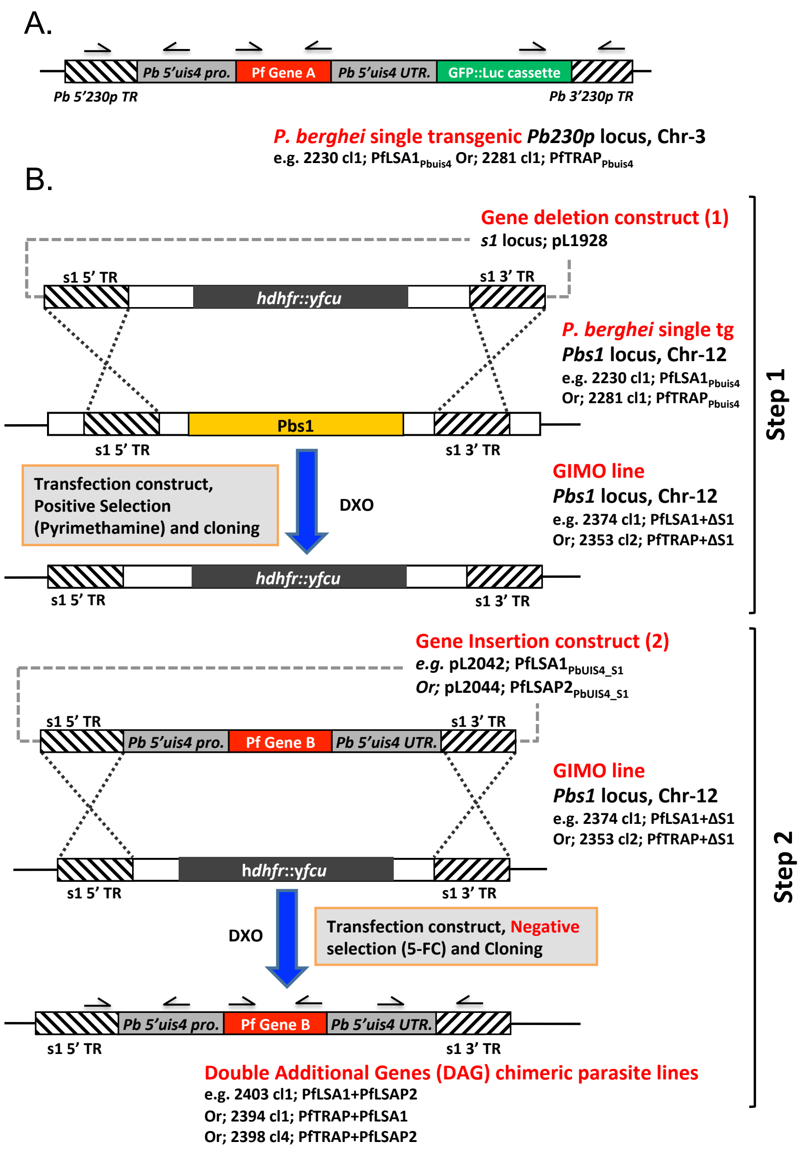

C.

Parent SAG line	GIMO line	Gene Insertion Construct	DAG chimeric parasite line
2230 cl1; PfLSA1$_{Pbuis4}$	2374 cl1; PfLSA1+ΔS1	pL2042; PfLSA1$_{PbUIS4_S1}$	2403 cl1; PfLSA1+PfLSAP2
2281 cl1; PfTRAP$_{Pbuis4}$	2353 cl2; PfTRAP+ΔS1	pL2042; PfLSA1$_{PbUIS4_S1}$	2394 cl1; PfTRAP+PfLSA1
2281 cl1; PfTRAP$_{Pbuis4}$	2353 cl2; PfTRAP+ΔS1	pL2044; PfLSAP2$_{PbUIS4_S1}$	2398 cl4; PfTRAP+PfLSAP2

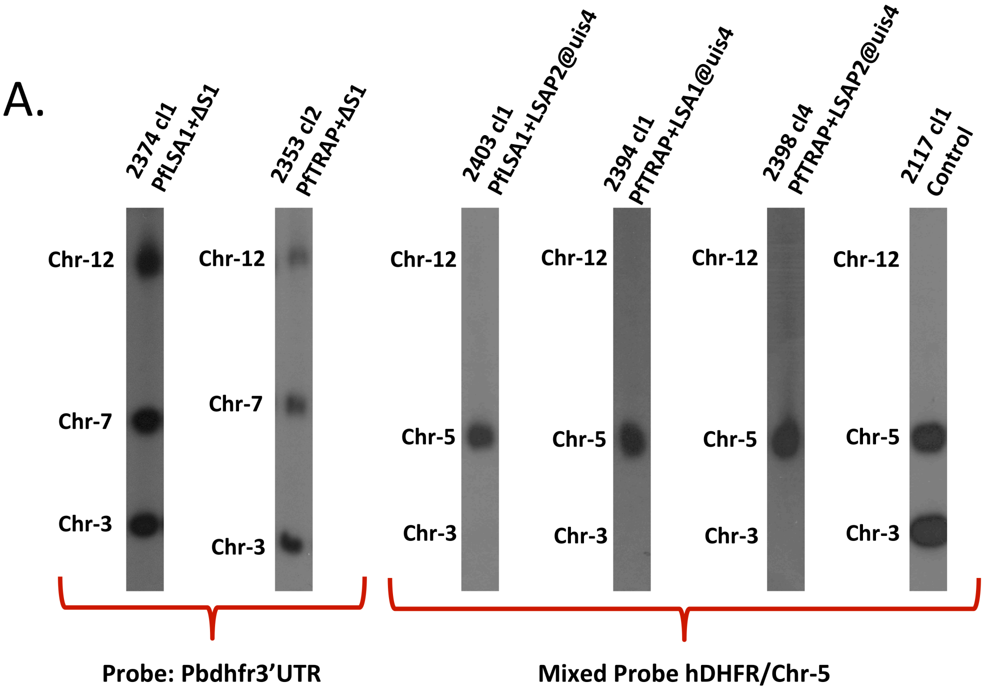

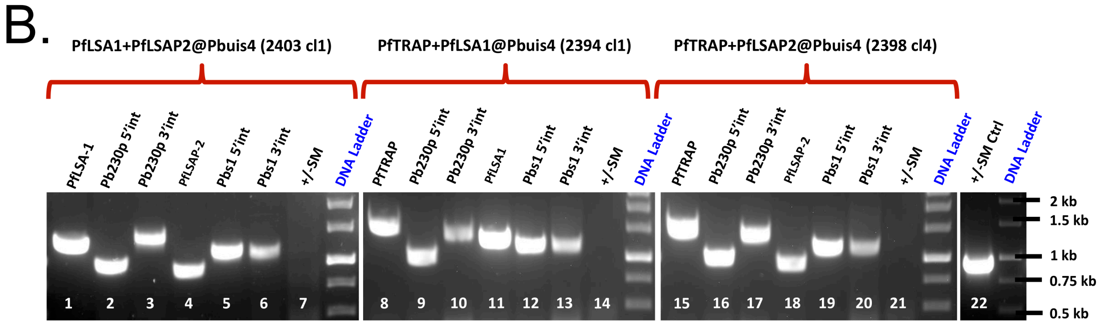

No	PCR product	Primers F	Primers R	Size (bp)
1	PfLSA-1	1086	1087	1.386
2	Pb230p 5'int	1080	1081	1.100
3	Pb230p 3'int	1051	1083	1.602
4	PfLSAP-2	1100	1101	1.023
5	Pbs1 5'int	1127	1081	1.309
6	Pbs1 3'int	1082	1128	1.308
7	+/-SM	1048	1049	NA

No	PCR product	Primers F	Primers R	Size (bp)
8	PfTRAP	1106	1107	1.727
9	Pb230p 5'int	1080	1081	1.100
10	Pb230p 3'int	1051	1083	1.602
11	PfLSA-1	1086	1087	1.386
12	Pbs1 5'int	1127	1081	1.309
13	Pbs1 3'int	1082	1128	1.308
14	+/-SM	1048	1049	NA

No	PCR product	Primers F	Primers R	Size (bp)
15	PfTRAP	1106	1107	1.727
16	Pb230p 5'int	1080	1081	1.100
17	Pb230p 3'int	1051	1083	1.602
18	PfLSAP-2	1100	1101	1.023
19	Pbs1 5'int	1127	1081	1.309
20	Pbs1 3'int	1082	1128	1.308
21	+/-SM	1048	1049	NA
22	+/-SM Control	1048	1049	927

Fig. S9: Genotype analysis of DAG chimeric parasites

Genotype analysis of the DAGs chimeric parasite lines PfLSA1+PfLSAP2@Pbuis4 (line 2403 cl1), PfTRAP+PfLSA1@Pbuis4 (line 2394 cl1) and PfTRAP+PfLSAP2@Pbuis4 (line 2398 cl4) and its intermediate GIMO mother-lines (2374 cl1 and; 2353 cl2).

(**A.**) Hybridisation of chromosomes (chr.) of lines 2374 cl1 and; 2353 cl2 (left panel) with the 3′UTR *Pbdhfr/ts* confirms integration of construct pL1928 (Fig. S2-A) into the *Pbs1* gene on chr. 12. In addition, this probe hybridizes to the GFP-Luc reporter-cassette in chr. 3 (Fig. S2-A) and to the endogenous *Pbdhfr/ts* on chr. 7. The correct integration of the second P. falciparum

expression construct (either: pL2042; or pL2044) (Fig. S2) into the *Pbs1* GIMO locus was confirmed by showing the removal of the h*dhfr::yfcu* SM cassette in the cloned chimeric parasite lines 2403 cl1, 2394 cl1, 2398 cl4 (right panel) . The southern blot was hybridized with a mixture of two probes: one recognizing h*dhfr* and a control probe recognizing chr. 5. As an additional control, parasite line 2117 cl1 was used with the h*dhfr::yfcu* SM integrated into chr. 3.

(**B.**) Genotype analysis by diagnostic PCR of the chimeric parasite lines 2403 cl1, 2394 cl1, 2398 cl4 confirmed correct integration of both first and second *P. falciparum* genes expression cassettes. Correct integration is shown by the absence of the h*dhfr*::y*fcu* SM, the presence of both first and second *P. falciparum* genes CDS, and the correct integration of the two different *P. falciparum* genes' constructs into the genome both at the 5' and 3'regions of Pb230 and Pbs1 loci, respectively (5'int and 3'int; see Fig. S2-B for primers' locations). Primers sequences used are shown in Table S1.

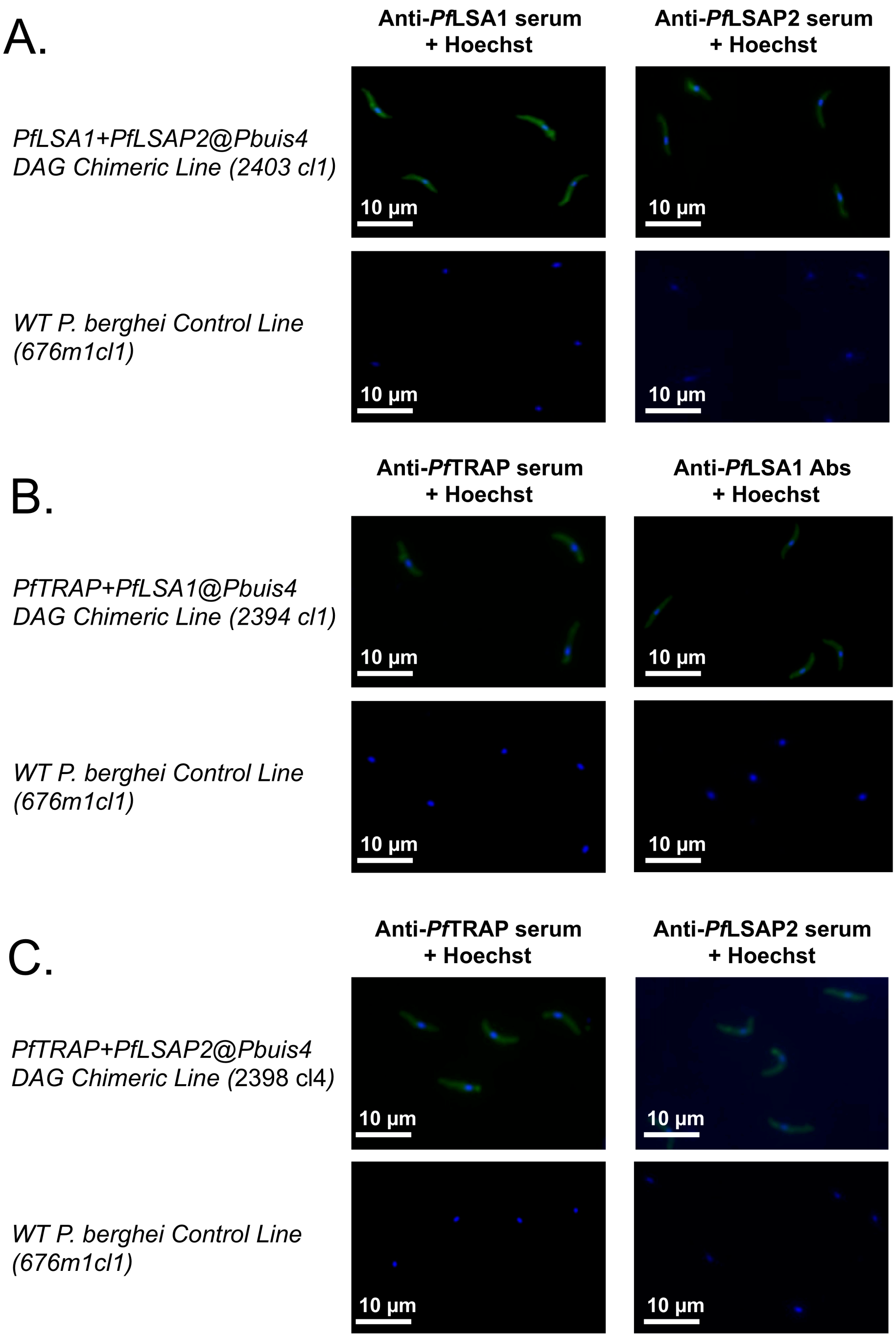

Fig S10: Confirmation of *P.falciparum* gene expression by IFAT staining of sporozoites

Expression of *P. falciparum* antigens on sporozoites of the DAG chimeric parasites were analysed by immunofluorescence- staining assay (IFA), using sera from mice vaccinated with the relevant viral vectors (Adeno followed by MVA). Purified sporozoites were fixed with 4% paraformaldehyde and blocked with 10% FCS+1%, prior to incubating with serum from vaccinated mice and detection of bound antibodies with the addition of Goat Anti-Mouse IgG-

Alexa 488. Nuclei were stained with 2% Hoechst-33342, prior to mounting slides with coverslips and image capture using a DMI-300B Leica microscope.

(**A.**) The DAG chimeric parasite; PfLSA1+PfLSAP2@Pbuis4 (line 2403 cl1), (**B.**) The DAG chimeric parasite PfTRAP+PfLSA1@Pbuis4 (line 2394 cl1) and (**C.**) DAG chimeric parasite PfTRAP+PfLSAP2@Pbuis4 (line 2398 cl4). The wild type *P. berghei* parasite (line 676m1cl1) has been used as a control in parallel to each DAG chimeric parasite.

Table S1: Primers for genotyping the DAGs chimeric parasite line 2403 cl1, 2394 cl1 and 2398 cl4

Primer No.	Description	Primer sequences
**1048**	hDHFR-yFCU (+/-SM) F	ATCATGCAAGACTTTGAAAGTGAC
**1049**	hDHFR-yFCU (+/-SM) R	CATCGATTCACCAGCTCTGAC
**1051**	Luciferase F	GTCGCCAGTCAAGTAACAAC
**1080**	Pb5'230p Integration F	ACTGTTATATTTGGTGATGGAATGG
**1081**	Pb5'230p Integration R	TATACATCCACGGATGCATAGAAG
**1082**	Pb3'230p Integration F	TCTGCATTAACCTTAAATATGAAAAACAC
**1083**	Pb3'230p Integration R	TTCAGTGAAATCGCAAACATAAGTATC
**1086**	PfLSA1 F	ATGAAGCACATCCTGTACATC
**1087**	PfLSA1 R	TCACAGCTTCATGAAGTACTTGG
**1100**	PfLSAP2 F	ATGTGGTTATGCAAAAGGGGACTG
**1101**	PfLSAP2 R	ATATATTTAATTTGTAACATTTCTCATGTTATTTTCTAAAG
**1106**	PfTRAP F	ATGAATCATCTTGGGAATGTTAAATATTTAGTC
**1107**	PfTRAP R	ATTTAATTCCACTCGTTTTCTTCAGG
**1127**	Pb3'S1 Integration R	TGTACTACTTTCACATCAAATTCAGTAACC
**1128**	Pb5'S1 Integration F	TAAAGGTTAGCATTCAATCTGTCTG
